# Independent and interactive effects of disease and methylmercury on demographic rates across multiple amphibian populations

**DOI:** 10.1038/s41598-025-99839-3

**Published:** 2025-05-19

**Authors:** Morgan P. Kain, Blake R. Hossack, Kelly L. Smalling, Brian J. Halstead, Daniel A. Grear, David A. W. Miller, Michael J. Adams, Adam R. Backlin, William J. Barichivich, Collin A. Eagles-Smith, Colleen Emery, Jillian E. Fleming, Robert N. Fisher, Elizabeth Gallegos, Duoa Jim Lor, Patrick M. Kleeman, Erin Muths, Ty Pan, Christopher A. Pearl, Charles W. Robinson, Catilin Rumrill, Brian J. Tornabene, J. Hardin Waddle, Susan C. Walls, Evan H. Campbell Grant

**Affiliations:** 1 Pennsylvania State University, State College, PA USA; 2 Eastern Ecological Science Center (Patuxent Wildlife Research Center), S.O. Conte Anadromous Fish Research Laboratory, U.S. Geological Survey, Turners Falls, MA USA; 3 U.S. Geological Survey, Northern Rocky Mountain Science Center, Missoula, MT USA; 4 Wildlife Biology Program, University of Montana, Missoula, MT USA; 5 U.S. Geological Survey, New Jersey Water Science Center, Lawrenceville, NJ USA; 6 U.S. Geological Survey, Western Ecological Research Center, Dixon, CA USA; 7 U.S. Geological Survey, National Wildlife Health Center, Madison, WI USA; 8 U.S. Geological Survey, Forest and Rangeland Ecosystem Science Center, Corvallis, OR USA; 9 U.S. Geological Survey, Western Ecological Research Center, Santa Ana, CA USA; 10 U.S. Geological Survey, Wetland and Aquatic Research Center, Gainesville, FL USA; 11 U.S. Geological Survey, Western Ecological Research Center, San Diego, CA USA; 12 U.S. Geological Survey, Western Ecological Research Center, Point Reyes Station, CA USA; 13 U.S. Geological Survey, Fort Collins Science Center, Fort Collins, CO USA

**Keywords:** Conservation biology, Population dynamics

## Abstract

Disease, alone or combined with other stressors such as habitat loss and contaminants, affects wildlife populations worldwide. However, interactions among stressors and how they affect demography and populations remain poorly understood. The amphibian chytrid fungus (*Batrachochytrium dendrobatidis*; Bd) is a sometimes-lethal pathogen linked with population declines and extirpations of amphibians globally. Laboratory evidence shows ubiquitous contaminants like methylmercury (MeHg) can reduce vigor and survival of amphibians, but population-level effects remain unclear. We used non-lethal sampling to assess how Bd and MeHg affected survival of juvenile and adult amphibians in 20 populations across the USA. Survival of several species declined with increasing Bd loads, including some species previously considered resistant to Bd (e.g., eastern newt [*Notophthalmus viridescens*]). Although our sampling for MeHg was less intensive than for Bd, we found MeHg can both directly reduce survival and synergistically magnify the effects of Bd infection. For a population of foothill yellow-legged frogs (*Rana boylii*), the estimated reduction in survival from MeHg exceeded that from Bd. Although effects varied widely among populations and species, our results help clarify the potential for synergistic effects of disease and contaminants and emphasize the complexity of identifying and quantifying the population-level effects of interactions among stressors.

## Introduction

Infectious diseases, whether alone or in conjunction with other stressors such as habitat loss and environmental contaminants, remain a growing threat to global biodiversity^1,2^. Effects from pathogens are often most detectable during epizootic stages characterized by high transmission, common infection, and high probability of mortality—conditions during which the mean infection intensity of a pathogen, and thus the probability of detecting infection, is expected to be highest^3^. Negative effects from pathogens are possible even in enzootic states^4^, however, with sublethal effects regulating population dynamics^1,5^. This makes quantifying host–pathogen dynamics during enzootic stages important^6,7^, especially in systems where interactions between disease and other stressors can amplify disease severity and potentially reduce the efficacy of interventions.

Chytridiomycosis is a lethal amphibian disease caused by the aquatic fungus *Batrachochytrium dendrobatidis* (Bd), which has contributed to catastrophic declines and extinctions of amphibian populations worldwide^8^. Most research into the effects of Bd on survival and population dynamics of amphibians in temperate North America has focused on species in the western United States, specifically mountain yellow-legged frogs (*Rana muscosa* and *R. sierrae*, collectively) and western toads (*Anaxyrus boreas*)^9,10^. These species experienced widespread declines, including population extirpations, coinciding with the first description of Bd in the late 20^th^ Century^11–13^. Despite some population recoveries, a recent synthesis of capture-mark-recapture (CMR) studies of four species (14 populations) of threatened or endangered ranid frogs (*Rana* spp.) in the western United States found that Bd-positive individuals had apparent survival probabilities 6–15% lower than Bd-negative individuals^10^. This work has revealed important insights about the transition from epizootic to enzootic systems (e.g.,^14^). For example, density-dependence in host–pathogen dynamics may have contributed to the persistence of some frog populations^7^. In fact, between 2000–2005, persistent populations of Sierra Nevada yellow-legged frogs (*R. sierrae*) were increasing in abundance, although still well below pre-Bd abundance^15,16^.

While research on Bd-host dynamics has naturally focused on species obviously affected by Bd, the pathogen infects most lentic-breeding amphibian species across North America, often without obvious signs of disease^17–21^. There is substantial variation in disease susceptibility among and within populations, even for species severely affected by chytridiomycosis^15,16,22,23^. Because of this variation, the trajectory of host–Bd dynamics varies from host extinction to persistence of host populations where effects on vital rates and populations are not obvious^14^. Even when a pathogen does not cause catastrophic declines, it may still affect populations by limiting population growth rates and increasing sensitivity to other drivers of decline. For example, even with positive population growth for reintroduced Sierra Nevada yellow-legged frogs, higher individual mortality rates attributed to Bd infection increased the probability of negative population growth with the introduction of secondary stressors^24^*.* Despite all the research effort devoted to understanding the nuanced effects of this pathogen, it remains a conservation-relevant example of a disease for which continued work is critical to understand causes of variation in population-level effects of infection.

Variation in the effects of Bd on populations is likely caused by several physical and ecological factors that may interact. Habitat degradation and the resultant exposure to contaminants such as mercury (Hg), chloride (freshwater salinization), and pesticides in freshwater systems is a threat to amphibian populations globally^25^. In particular, Hg is a contaminant of global concern that poses a unique risk to wildlife populations, including amphibians, largely because of its ubiquity. Human activities such as fossil fuel production, mining, and industrial applications are primary sources of environmental Hg^26^, and wetland sediments can serve as a reserve of Hg that under appropriate conditions can be converted to methylmercury (MeHg;^27,28^). MeHg is more bioavailable and toxic compared to elemental Hg and can biomagnify in aquatic and terrestrial food webs^8^.

Methylmercury can have both lethal and sublethal effects on amphibians^29–31^, though there remains a general lack of evidence translating laboratory-derived effects into effects on survival, population growth, or abundance in free-ranging populations^32,33^. Amphibians bioaccumulate MeHg from the environment^34–36^, which may cause direct toxicity by altering physiological functions, disrupting endocrine function, and interfering with hormonal signals of metamorphosis^29,37^. It can also weaken immune responses, potentially increasing risk from infectious disease^38–40^, though we are unaware of any studies assessing the interaction between MeHg and Bd.

**Fig. 1:**
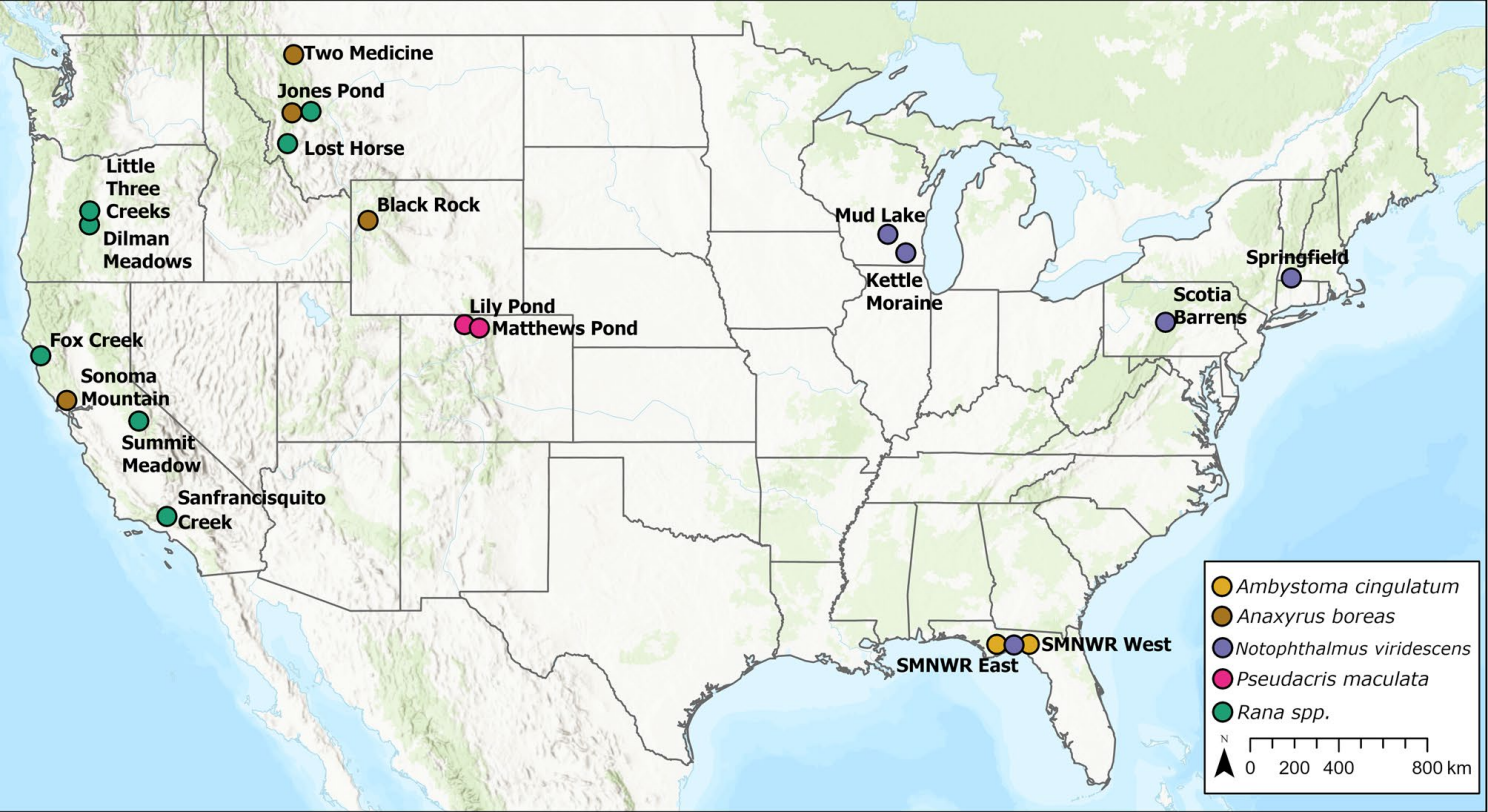
Study sites used to estimate independent and interactive effects of amphibian chytrid fungus (Bd) and methylmercury (MeHg) on survival of amphibians across the contiguous USA. The sites represent 20 populations from 10 species of amphibians. Species included frosted flatwoods salamander (*Ambystoma cingulatum*), western toad (*Anaxyrus boreas*), eastern newt (*Notophthalmus viridescens*), boreal chorus frog (*Pseudacris maculata*), and 6 ranid frog species (*Rana* spp.). The 6 ranid frog species (and site location names) were: Columbia spotted frog (*Rana luteiventris*; Jones Pond, Lost Horse), Oregon spotted frog (*R. pretiosa;* Dilman Meadows), foothill yellow-legged frog (*R. boylii;* Fox Creek), California red-legged frog (*R. draytonii;* San Francisquito Creek), Sierra Nevada yellow-legged frog (*R. sierrae;* Summit Meadow), and Cascades frog (*R. cascadae;* Little Three Creeks). Map made using ArcGIS Pro 3.4.0 (https://www.esri.com/en-us/arcgis/products/arcgis-pro/overview; under copyright https://pro.arcgis.com/en/pro-app/latest/get-started/copyright-information.htm). Baselayer sources: Esri, Garmin, Food and Agriculture Organization, National Oceanic and Atmospheric Administration, U.S. Geological Survey, Environmental Protection Agency.

Here we present results from longitudinal, capture-mark-recapture studies of 20 populations of 10 amphibian species across the continental USA (Fig. 1). This study is the most extensive to date for directly estimating effects of enzootic Bd on demography of amphibian populations, and the first to estimate the additive and interactive (with Bd) effects of MeHg on survival of free-ranging amphibians. Our work provides new insights into how enzootic Bd is affecting amphibian survival in temperate North American amphibian populations. Further, rather than focus solely on known Bd-susceptible species and populations, we provide results for species with prior evidence of susceptibility to Bd, as well as common species for which the effects of Bd on survival were previously unknown.

## Results

### Effects of *Bd* on survival

**Fig. 2:**
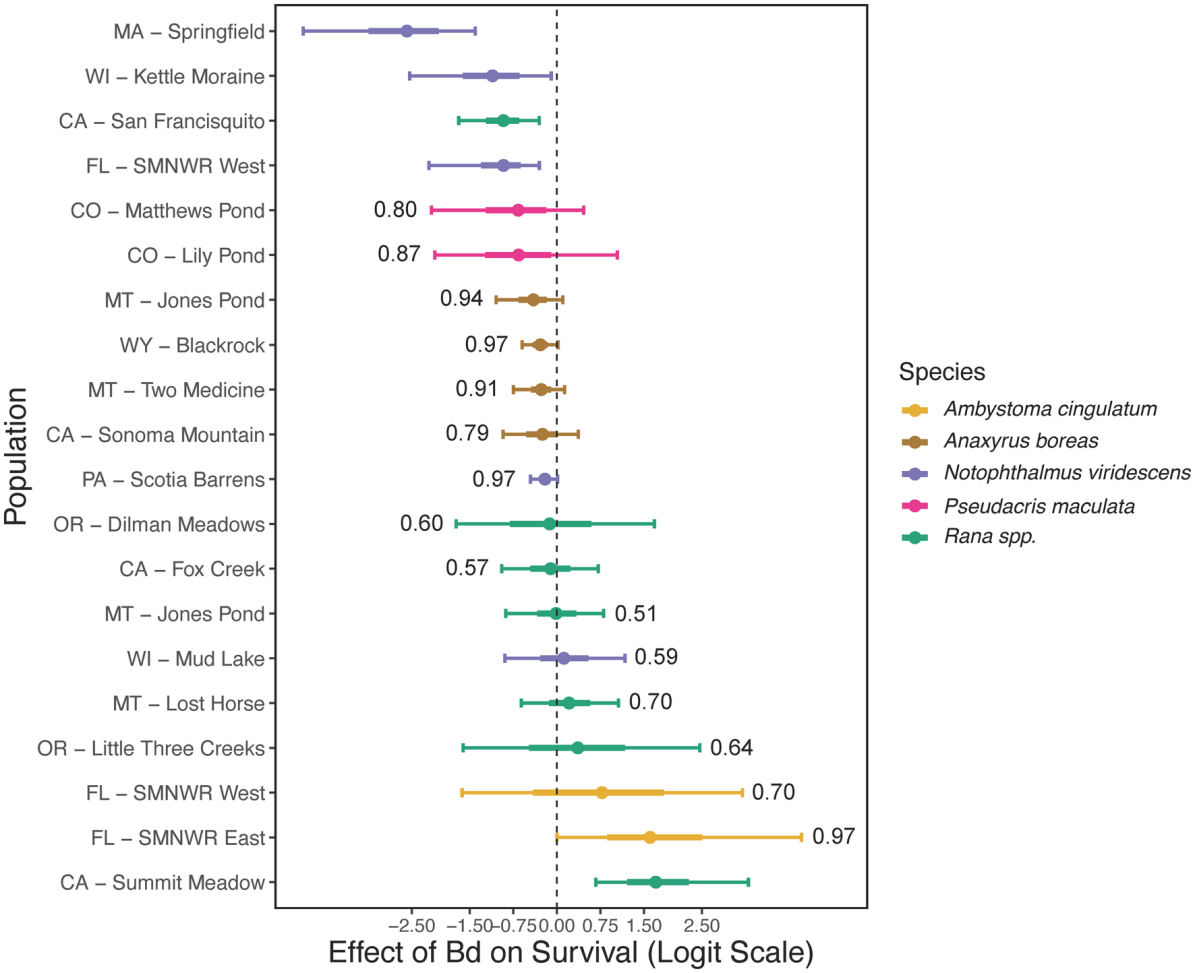
Coefficient estimates for the effect of estimated yearly individual Bd loads on individual apparent survival between years. Estimates are presented on the logit scale using scaled Bd. Points show medians of posteriors. Text next to CI overlapping zero gives the proportion of the posterior greater or less than zero. Wide intervals show 95% CI while the internal (thick) lines show 60% CI. The *Rana* species and their populations are, from top to bottom: CA-San Francisquito: *R. draytonii*; OR-Dilman Meadows: *R. pretiosa*; CA-Fox Creek: *R. boylii*; MT-Jones Pond: *R. luteiventris*; MT-Lost Horse: *R. luteiventris*; OR-Little Three Creeks: *R. cascadae*; CA-Summit Meadow: *R. sierrae*. For estimates on a probability scale see Figure S2. See Fig. 1 for locations of each population.

Using our 2 CMR models fit to mark recapture data for western toads (*Anaxyrus boreas*), eastern newts (*Notophthalmus viridescens*), boreal chorus frogs (*Pseudacris maculata*), frosted flatwoods salamanders (*Ambystoma cingulatum*), and 6 ranid frogs, we were able to determine how Bd affected annual survival of individuals. We found moderate evidence that survival of individual western toads, eastern newts, and California red-legged frogs (*R. draytonii*) decreased when infected with Bd, as evidenced by 95% credible intervals (CI) below zero or barely overlapping zero for most or all populations of these species (Fig. 2). Estimates can be placed into 5 categories based on the values of the point estimate (median), overlap of 95% CI with zero, and width of 95% CI: (1) clearly detectable negative effects (95% CI not overlapping zero; eastern newt: MA [Springfield], WI [Kettle Moraine], FL [SMNWR East]; California red-legged frog: CA [San Francisquito]); (2) moderate evidence for a negative effect (95% CI overlapping zero but median below zero) all 4 populations of western toad; eastern newt: PA [Scotia Barrens]; both populations of the boreal chorus frog); (3) no clear effect and high uncertainty (many ranid populations and a second WI eastern newt population [Mud Lake], see Fig. 2); (4) moderate evidence for a positive effect (95% CI overlapping zero but median above zero: Cascades frog (*R. cascadae*), OR [Little Three Creeks]; eastern newt, FL [SMNWR_E]; both populations of the frosted flatwoods salamander); and (5) statistically clear evidence of a positive effect (Sierra Nevada yellow-legged frog: CA [Summit Meadow]). Credible intervals were especially wide in populations with few recaptures or few swabbed individuals (e.g., populations of boreal chorus frogs and frosted flatwoods salamanders; see Fig. 2, Table S1, and Figure S1).

**Fig. 3:**
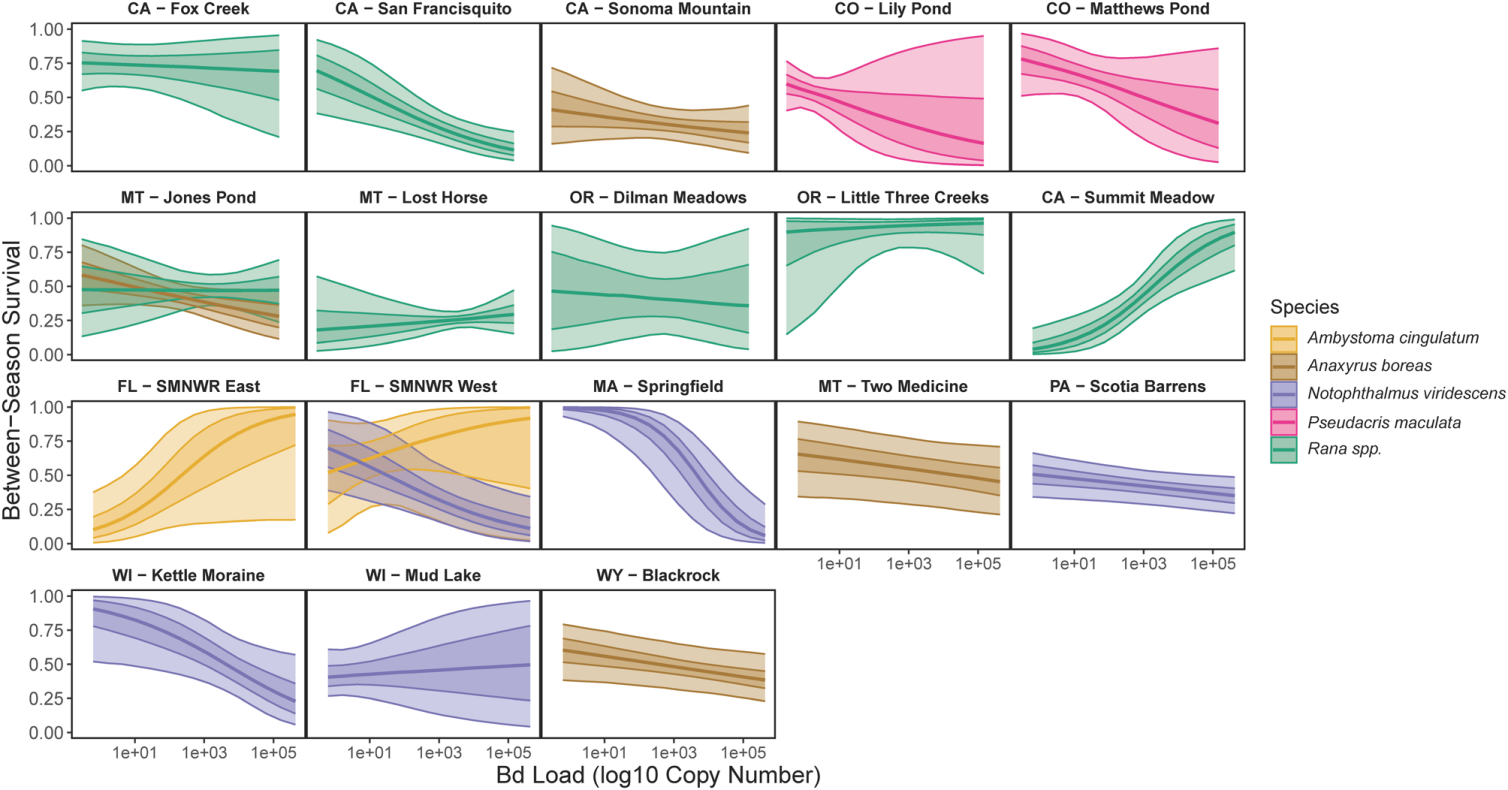
Between-year survival of individuals in all 20 populations depending on their yearly average Bd load (x-axis). Bold lines give median estimates, darker envelopes show 60% CI, faint envelopes show 95% CI.

High uncertainty for yearly survival of ‘average’ individuals in most populations (i.e., those with a mean yearly Bd load, snout vent length (SVL), and MeHg concentration; Figure S2) limited conclusions regarding between-year survival in most populations (Fig. 3, Figure S3). However, eastern newts in general seemed especially sensitive to a 1 standard deviation increase in Bd load (which equates to the addition of ~ 4 Bd load units on the log scale), as were California red-legged frogs from the San Francisquito, CA, population (Figure S3). The proportional decrease in survival given a 1 standard deviation increase in Bd load was also high for boreal chorus frogs in Colorado, but the estimated effect was highly uncertain. Estimated population-level Bd load distributions for all 20 populations that underlie these predictions reveal the following: high loads in all eastern newt populations apart from the Wisconsin (WI) Mud Lake population, high means but wide distributions for western toads, and substantial variation among species and populations of ranid frogs (Figure S4). We did not detect Bsal in any sample.

### Independent and interactive effects of MeHg on survival

**Fig. 4:**
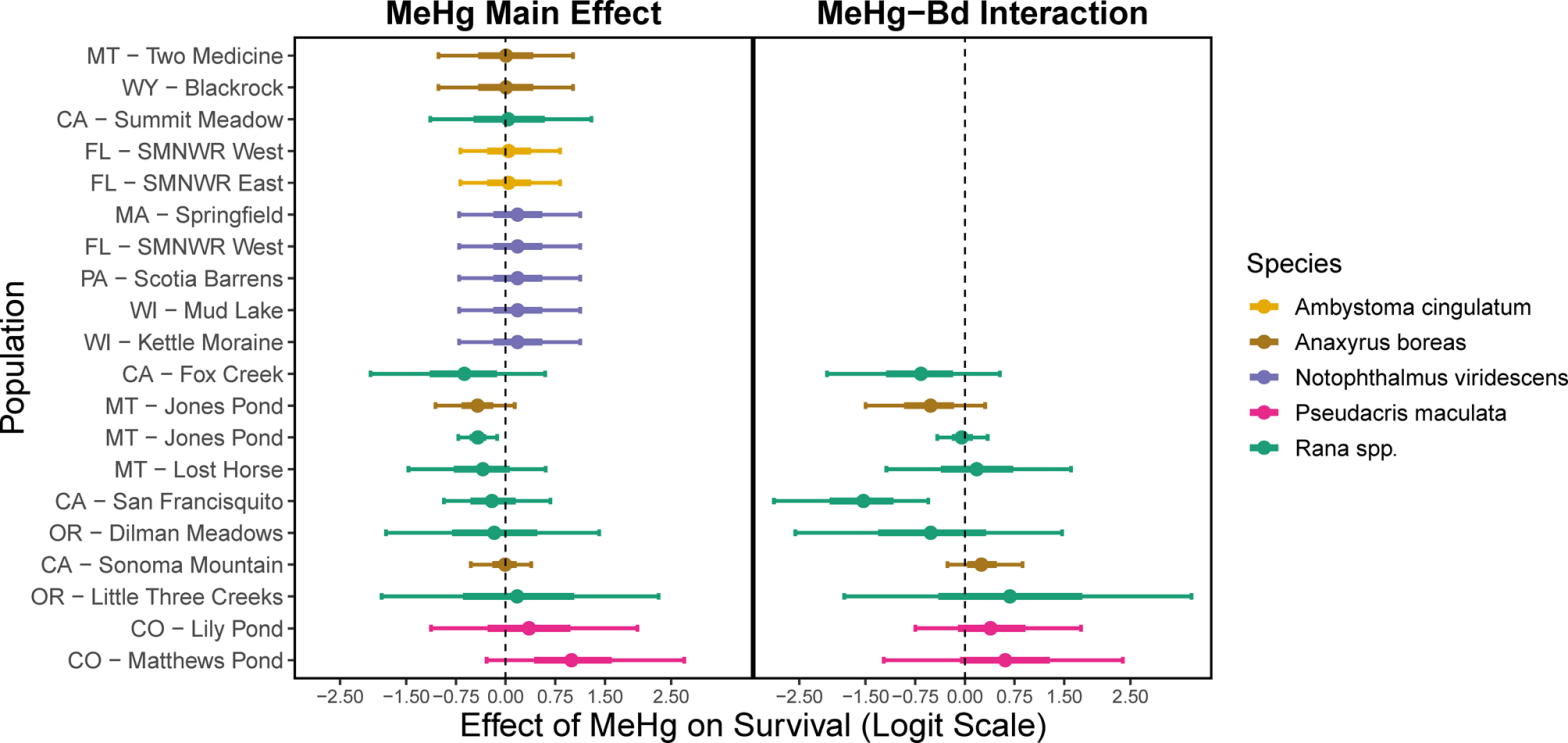
Model estimates for the main effect of MeHg concentration (left) and the interactive effect of individual-level Bd loads and MeHg concentrations (right) on individual between-year survival. Points show the median of the posterior distributions, wide intervals show 95% CI, while the internal (thick) lines show 60% CI. Estimates for the main effect of MeHg in populations with an estimated MeHg-Bd interaction effect were obtained from our primary model, while estimates for the main effect of MeHg in populations without an estimated MeHg-Bd interaction effect (e.g., western toads [Two Medicine]) were obtained from our secondary model that estimated population-level MeHg and not individual-level MeHg (see supplemental material).

Direct effects of MeHg on survival, and the effects of an interaction between MeHg and Bd on survival, were estimated with large uncertainty for most populations (Fig. 4). Notable exceptions included a clear, negative additive effect of MeHg on survival of Columbia spotted frogs at Jones Pond in Montana (MT). There was also a synergistic, negative effect of Bd and MeHg on survival of California red-legged frogs, and a negative additive effect of MeHg on survival (88% of the posterior), with evidence for synergistic effects with Bd (80% of the posterior) on survival of western toads at Jones Pond in MT. We found a slightly positive relationship between survival and MeHg at Matthews pond boreal chorus frogs, though the credible interval overlapped zero. A summary of the data for MeHg concentrations among individuals in the 16 populations in which MeHg was measured can be found in Figure S5.

### Other correlates of survival

Larger individuals (as measured by snout-vent length; driven primarily by age but also local conditions) had greater between-year survival in most populations; the estimated effect of length was strong for many species, including the frosted flatwoods salamander, eastern newt, and ranid frogs (Figure S6). Individuals in all species and populations were estimated to have high within-year (between primary period) survival (15/20 populations > 90%, all populations > 50%), but with high uncertainty (Figure S7).

### Detection

Females were more difficult to detect than males (estimated collectively across all species; median: -0.33, 95% CI: -0.39, -0.27 [coefficients on the logit scale]), which results partly from different breeding behavior and availability for capture. There was some evidence that decreasing water levels of sites across a survey period increased detection probability, and increasing amounts of vegetation decreased detection probability, though 95% CI for both estimates overlapped zero (drawdown: median: 0.14, 95% CI: -0.01, 0.29; vegetation: median: -0.078, 95% CI: -0.43, 0.25 [estimates on the logit scale]). Credible intervals for all species-specific random deviates for detection overlapped zero; most of the variation in detection was captured by the site and primary period-by-sampled-subsites random effect terms (Figure S8).

### Population sizes

We estimated relatively consistent or slow declines in population sizes for most populations across the 4 to 5 years that each was monitored (Figures S9, S10). Among all ranids, for example, we estimated mostly consistent population sizes in 5 populations and minor declines in the Oregon spotted frog [Dilman Meadows] and California red-legged frog [San Francisquito] populations (Figures S9, S10). Among the other species (Figures S9, S10), 2 populations stand out as exceptions: the WI eastern newt [Mud Lake] population was estimated to be increasing, and a MT western toad [Two Medicine] population was estimated to be decreasing. Both populations experienced consistent numbers of overall captures each year (2018‒2022); however, detection probability was estimated to be lower in later years in the WI eastern newt population, leading to higher population size estimates. An opposite trend in detection for the MT western toad population lead to an estimate of decreasing population size.

## Discussion

Ecologists have long recognized that many drivers of amphibian decline likely interact (e.g.,^41–43^), but it is challenging to obtain quantitative estimates for additive and synergistic drivers of demographic rates from data collected in free-ranging populations. The Bd panzootic has generated substantial variation in host‒Bd population infection and survival dynamics across both imperiled and common species^10,14^. Similarly, while MeHg is expected to negatively affect individuals^29–31^, large spatial variation in MeHg exposure^34,36^ alongside variation in sensitivity among species and ecological contexts produces a highly complex Bd‒MeHg threat landscape. Similarly, while MeHg exposure is expected to negatively affect individuals^29–31^, large spatial variation in MeHg exposure^34,36^ alongside variation in sensitivity among species and ecological contexts likely also fosters a highly complex Bd‒MeHg threat landscape.

### Effects of Bd on survival

For Bd, we found load-dependent reductions in survival for some host species with well documented Bd-related declines (e.g., western toads). We also detected negative effects of Bd in multiple populations and species for which Bd has not historically been a concern (Figs. 2, 3). Although it was not a focus of this study, as we did not optimize our model for estimating changes in population size, we found some evidence for population declines in many populations over the study period (Figures S9, S10). Correlations between changes in population size (Figures S9, S10) and Bd effects (Fig. 2) within our populations were weak overall, though a few select populations with moderate estimated declines—namely the California red-legged frog population in CA (San Francisquito) and western toad population in MT (Two Medicine)—had moderate to large estimated negative effects of Bd on individual survival. Alternatively, the 2 populations of eastern newts with the largest estimated negative effects of Bd showed stable populations. The variation in effect sizes and uncertainty within and among populations and species highlight that identifying the relative roles of disease and other stressors in population dynamics remains a complex ecological problem.

Variation in the effects of Bd on individuals, and uncertainty about its population-level effects, has characterized much of the research on this globally important pathogen. Some of this variation is likely attributable to phases of the disease pandemic (e.g., endemic vs. epidemic), environmental variation that affects both pathogen and hosts, genetic diversity and strains of Bd, and population- and species-level variation in susceptibility to the disease^10,14,23,44^. We similarly found a wide range of effects of Bd on survival both within and across species. Five of our 6 most-negative effects were for eastern newts and boreal chorus frogs (Figs. 2, 3), species that have not previously been associated with widespread concern over sensitivity to Bd. The next 4 most-negative mean estimates were for western toads (Fig. 2), which reinforces previous findings that Bd reduces survival and has contributed to widespread declines of that species^10,12^.

Negative effects of Bd on survival for four of five eastern newt populations and both boreal chorus frog populations, and their general high sensitivity to increased Bd loads (Figs. 2, 3, S4), was surprising given that these species are common and widespread with little prior concern about their vulnerability to Bd. For example, eastern newts are considered to be common and abundant across our study areas, despite detections of high Bd loads in populations across the eastern United States (e.g.,^45–48^). It was thought that eastern newts should be resistant to Bd because of abundant skin bacteria that can inhibit Bd growth^49^. Apart from the Mud Lake, WI, population, which had among the lowest estimated Bd loads, eastern newts had the second-highest average loads of species we studied (western toads had the highest; Figure S4). The two eastern newt populations with the overall largest estimated negative effects of Bd (Springfield, MA, and Kettle Moraine, WI) had the second- and fourth-highest average estimated Bd loads of the 20 populations (Figure S4). It is unclear if eastern newt and boreal chorus frog populations are threatened by Bd. However, high prevalence and loads of Bd in eastern newt populations could have broader community implications for disease risk given that eastern newts could serve as a form of amplifying host. Eastern newts are highly aquatic, typically abundant, and have high terrestrial mobility that may combine to play a large role in intra- and inter-pond disease dynamics. The negative effect of Bd on survival of eastern newts and boreal chorus frogs, despite high population abundances, illustrates the sometimes surprising variation in outcomes of Bd infection.

For ranid frogs (*Rana* spp.) in the western USA, the effect of Bd on survival was estimated to be near zero for all but 2 populations (San Francisquito California red-legged frog population, negative effect; and Summit Meadow Sierra Nevada yellow-legged frog population, positive effect). However, high uncertainty around these estimates limits conclusions. These results differ from an earlier analysis that showed Bd reduced the survival of ranid frogs across populations in the western USA, including earlier sampling and longer time-series of data from some of the same populations included in the current analysis^10^. On one hand, these populations may in fact be minimally affected by Bd, perhaps because of evolved resistance or tolerance (Carvalho et al., 2024). On the other hand, Bd could be reducing survival within these populations and our estimates are simply too uncertain to draw strong conclusions. Also, ranid frogs had among the lowest estimated apparent survival at average Bd loads (Figure S2), which could have obscured some of the effects of Bd. Across all species in our study, larger individuals also had greater between-year survival in most populations — a 1 SD increase in length had a larger effect on survival than a 1 SD increase in either Bd or MeHg (Figure S6). The estimated effect of length was especially strong for the frosted flatwoods salamander, eastern newt, and ranid frogs, which could have further obscured effects of other stressors on survival.

### Independent and interactive effects of MeHg on survival

Contaminants are often hypothesized to interact with other stressors, including disease, but research on these interactive effects remains inconclusive and limited^50–52^. Our results revealed that MeHg can both directly reduce survival of individuals and synergistically reduce survival of individuals in combination with Bd infection (Fig. 4) in some populations. The 3 largest estimated negative effects of MeHg on survival were for Columbia spotted frogs at Jones Pond, MT (for which the 95% CI did not overlap zero), foothill yellow-legged frogs (*R. boylii*) at Fox Creek, CA, and western toads at Jones Pond, MT (Fig. 4). A broadscale assessment of MeHg concentrations in 26 amphibian populations (14 species; many of these populations are included in the current study) across the USA during 2017–2021 showed whole-body MeHg concentration for Columbia spotted frogs at Jones Pond and foothill yellow-legged frogs at Fox Creek were higher than for most other populations^36^, while western toads at Jones Pond had the lowest mean whole-body concentrations of the 4 western toad populations sampled^36^. In fact, survival in the Fox Creek population of foothill yellow-legged frogs was estimated to be more negatively impacted by MeHg than Bd (difference of -0.63 in median estimates on the logit scale) (Fig. 1, Fig. 4).

There is a general lack of evidence translating laboratory derived effects of many contaminants (including Hg) into effects on survival, population growth, or abundance in wild populations^32,33^. We focused on juvenile and adult amphibians because those life stages typically contribute the most to population growth, more directly linking our results with expected population demographics^53,54^. Our study provides clear evidence of a MeHg‒Bd interaction in one population (California red-legged frog*,* San Francisquito, CA) and moderate evidence in 2 populations (foothill yellow-legged frogs at Fox Creek, CA; western toad at Jones Pond, MT). For most populations, we saw no clear effect on survival in our Hg‒Bd interaction models. Notably, however, clear evidence of negative effects of MeHg on survival of 2 species at a single pond (Jones Pond, MT) was not because MeHg resulted in a greater reduction in survival compared to other locations, but instead because intensive sampling of those populations allowed for more precise estimates compared to most other study populations. The substantial variation in apparent sensitivity or vulnerability to MeHg suggests it will not be simple to infer effects of MeHg from tissue concentrations alone, because effects may be mediated by innate or adaptive tolerance, exposure, environmental differences, natural history differences in habitat preferences, or other stressors such as pathogens. Though our work does not directly fill the need for estimates on the relationship between MeHg and population growth or abundance, these initial field-based estimates on survival of adults moves us closer to understanding the potential population-level effects of MeHg.

Several uncertainties also limit our conclusions about how interactions between Bd and MeHg affected survival of amphibians. First, for most populations, estimates of the interaction of Bd and MeHg were highly uncertain with no clear evidence of a negative effect (i.e., wide 95% CIs overlapping zero: Fig. 4); this resulted partly because the best coverage of individual-level MeHg measures were for ranid frog species, for which the effect of Bd was difficult to estimate (as discussed above). Second, variation in exposure to other unmeasured contaminants (e.g., other metals, per/polyfluoroalkyl substances (PFAS), pesticides) among populations could have confounded the overall effect of MeHg. Third, we were unable to estimate individual-level effects of MeHg on survival or MeHg‒Bd interactions for many species, including eastern newts. The eastern newt populations we sampled experienced the greatest reductions in survival from Bd, but limited sampling for MeHg (see Table S1 for sampling coverage) restricted our ability to estimate effects of MeHg alone or in combination with Bd. These limitations emphasize the need for more studies to investigate interactions across a range of species and MeHg concentrations and further field investigations with more intensive sampling of populations that are potentially susceptible to Bd. This type of sampling is now even more practical with recent validation of non-lethal methods (a single toe- or tail-clip) to provide an adequate measure of MeHg in adult amphibians^50–52^. Using non-lethal methods can facilitate future studies on the effects of MeHg even for rare or federally protected species, for which the collection of large numbers or lethal collections are not advised nor allowed.

### Bd dynamics and modeling framework

Seasonal dynamics of the intensity of Bd infection in individuals may be an underappreciated phenomenon, but likely represents an important dynamic across many host-Bd systems^55,56^. Species life-histories (e.g., brief congregations at breeding sites) and logistics of site access limited sampling for many populations to a single visit (Table S1). Without multiple observations of individuals within a season, we were unable to fit an explicit model of infection dynamics and Bd growth rates to directly estimate individual-level infection probabilities. To accommodate individual variation in Bd load within a sampling season without replicate observations and an explicit dynamics model, we developed the model with a random effects framework that estimated a single mean value for every individual in each year. This approach leverages the regularization behavior of the random effects framework to pull extreme-value individuals with few measures towards the global mean, as a method of phenomenologically approximating the biological expectation that an individual with a single measure of zero load in a population with a high disease prevalence will likely experience a non-zero load at some point in the year. For more details on our Bd modeling approach see the online supplemental material.

An alternative hypothesis to explain the enzootic dynamics (e.g., small estimated impacts of Bd on survival and stable populations) we observed is the deeper co-evolutionary history between amphibian populations and the Bd global pandemic lineage (GPL) genotypes that evolved in North America^44,57^ compared to dynamics produced by more recent invasions. This hypothesis has emerged from several intensive genetic studies of Bd in North America that revealed high genetic diversity among multiple divergent GPLs in multiple regions infecting multiple host species^18,44,57^. We do not know the genetic identity of Bd infecting our study populations, but these studies suggest infection by the GPLs is likely. Understanding the latent interactions between Bd genotypes, host species, invasion, and co-evolution that result in the variety of demographic effects requires much more attention because the current landscape of Bd is marked by human-mediated, global mixing of divergent Bd lineages^44,58^.

## Conclusions

Amphibian chytridiomycosis remains an important driver of survival and population dynamics, even in populations where Bd has been present for > 20 years (e.g.,^7,10,12^). Our coordinated sampling effort across the contiguous USA, using a multi-species, multi-population, and multi-year modeling effort, provides robust results of the on-going effects of Bd on amphibians. Our results add further evidence that Bd is still limiting population growth and abundance for many species, even in the absence of epizootic events or catastrophic declines. Our results also highlight the dangers in making assumptions that species that have not suffered obvious declines linked with Bd (e.g., eastern newts, boreal chorus frogs) are not vulnerable to chytridiomycosis. effects of Bd were load-dependent and varied among species and sites, and local environmental factors such as MeHg exposure contributed to variation in responses. Quantifying how a common contaminant like MeHg can both directly reduce survival of individuals and synergistically magnify the effects of Bd adds clarity in understanding multiple threats while also highlighting the complexity involved in estimating the combined effects of disease and contaminants on survival and other demographic rates of populations. These results also highlight the challenges of managing populations in the face of endemic disease and other environmental stressors.

## Materials and methods

### Field sampling

Capture-mark-recapture (CMR) data were collected from 20 amphibian populations across the contiguous USA during 2017–2021 (Fig. 1). We define a population as individuals of a single species from a single aquatic site. Some sites were comprised of ‘subsites’, where one or more areas of a larger waterbody, or single ponds within a matrix of waterbodies, were within the expected seasonal movement capability of the target species. The 10 species in our dataset included the western toad (*Anaxyrus boreas*, n = 4 populations); eastern newt (*Notophthalmus viridescens*, n = 4); boreal chorus frog (*Pseudacris maculata*, n = 2); frosted flatwoods salamander (*Ambystoma cingulatum*, n = 2); Columbia spotted frog (*Rana luteiventris*, n = 2 ); and one population each of 5 other *Rana* spp.: Oregon spotted frog (*R. pretiosa*), foothill yellow-legged frog (*R. boylii*), California red-legged frog (*R. draytonii*), Sierra Nevada yellow-legged frog (*R. sierrae*), and Cascades frog (*R. cascadae*). Three of the 6 ranid species are listed as federally threatened or endangered (*Rana sierrae*: endangered; *R. pretiosa* and *R. draytonii*: threatened; *R. boylii*: status varies throughout range) and *A. cingulatum* is listed as federally threatened in the USA.

All populations were sampled following a standardized robust-design CMR protocol^59^. Briefly, animals were sampled during ≥ 1 primary periods per year when populations were assumed closed (separated by multiple weeks to 1 year depending on the frequency of monitoring), each of which consisted of ≥ 1 secondary period separated by a few days or less (see Table S2 and Figure S11 for all sampling occasions in all populations and Table S3 for capture numbers). During sampling events, we marked captured individuals with passive integrated transponder (PIT) tags or elastomer and recorded their length, sex, and life stage (juvenile or adult). Most captured individuals were swabbed for Bd at least once per primary period, though in some populations a subset of individuals recaptured multiple times in the same primary period were re-swabbed.

A subset of individuals was also sampled for MeHg in most populations (see Table S1 for a summary of Bd and MeHg sampling by population). We estimated whole-body MeHg concentrations from a single toe for frogs and toads (generally posterior toe L4) or a ~ 2-cm piece from the tail of salamanders. Further details on trapping strategies, handling methodologies (Bd swabbing and tissue sampling), and animal care and use documentation for all populations are in the Supplemental Information. All experimental protocols were approved by a named institutional or licensing committee (see Table S4 for details), and all methods were carried out in accordance with relevant guidelines and regulations, and in accordance with ARRIVE guidelines. All raw data are available at DOI: 10.5066/P9LSR4HY^64^.

### Laboratory methods

We extracted Bd DNA from swabs with slight modifications to the protocol described by Boyle et al.^60^. Extracted DNA was analyzed for Bd using a real-time TaqMan polymerase chain reaction (PCR) as described in Blooi et al. 2013^61^ for duplex detection of Bd and *B. salamandrivorans* (Bsal). We report all intensity data in units of estimated target copy number, as opposed to zoospore equivalents, because the number of PCR target ITS sequence copies varies by Bd lineage and the distribution of Bd genetic lineages is not well characterized across the USA^62^. For MeHg analysis, samples were dried to a constant mass and, depending on dry mass, analyzed whole or homogenized to a fine powder in a glass vial and analyzed following EPA Method 1630^63^. After laboratory analysis, toe or tail MeHg concentrations were converted to whole-body MeHg concentrations using 2 regression equations derived from prior validation data^36,64^. More details about laboratory methods for Bd and MeHg can be found in Supplemental Information (Supplemental Methods: Laboratory Methods).

### Models

To analyze the CMR data, we developed 2 hierarchical Bayesian CMR models that built on the standard Cormack-Jolly-Seber (CJS) model^65–67^ and differed in the complexity of the MeHg part of the analysis. Both models incorporated within- and between-year pathogen dynamics for hosts. The first model was based on species and sites with higher quality MeHg data and estimated individual-level MeHg for individuals when not sampled. The second model estimated only population average MeHg concentrations because of a lack of sufficient individual-level MeHg measures in several populations. We used CMR models because they allow demographic parameters to be estimated while accounting for various sources of uncertainty, including imperfect detection and incompletely observed covariates^68,69^.

Both models built on a simple CJS specification in 3 specific ways. First, to simultaneously analyze data from multiple populations, each model included a combination of fixed and random effects to model variation among the unique amphibian species and populations fit with that model. Second, we incorporated latent variables and latent process models for variables that could not be observed perfectly, or that were only observed when animals were captured, including individual snout-to-vent length (SVL) and Bd infection intensity (e.g., the nature of our data collection means that we only observed Bd infection intensity if an animal is both captured and swabbed). Specifically, both models estimated within-year individual-specific average Bd infection intensities, allowing this value to vary across both years and populations. We separately modeled within-season processes (survival between primary periods within a single year) and across-season survival (survival from the last primary period in one year to the first primary period the next year). We modeled survival as *apparent survival*, which estimates if an animal left a population but does not separate mortality from permanent emigration.

We introduce our primary model here in brief; further detail describing how our models built upon existing CJS models can be found in the Supplemental Information (Supplemental Methods: Models). We also describe the ways in which our secondary model (which considered only population-level MeHg concentrations) differed from our primary model in the Supplemental Methods: Models.

We used the CJS model to estimate between-year survival$\left( {\varphi_{\left( B \right)} } \right)$, within-season survival $\left( {\varphi_{\left( W \right)} } \right)$, and the effects of sex, size, Bd load, and MeHg on survival. We made judicious use of hierarchical structures to inform parameters across populations while obtaining population-specific estimates of mean sex-specific apparent survival and the effects of variables on survival. Our model for between-year survival (the final primary period in year *y* to the first primary period in year *y* + *1*) for populations with sufficient MeHg data was:

1 $$\begin{gathered} \left( {\varphi_{\left( B \right)i,s,j,y} } \right) = \underbrace {{\alpha_{s} + \alpha_{x} + a_{j} }}_{Intercept} + \underbrace {{\left( {\beta_{{Bd_{s} }} + b_{{Bd_{j} }} } \right)Bd_{i,s,j,y} }}_{BdEffect} + \underbrace {{\left( {\beta_{{MeHg_{s} }} + b_{{MeHg_{j} }} } \right)MeHg_{i,s,j,\left[ y \right]} }}_{MeHgMainEffect} \hfill \\ + \underbrace {{\left( {\beta_{{Bd - MeHg_{s} }} + b_{{Bd - MeHg_{j} }} } \right)Bd_{i,s,j,y} MeHg_{i,s,j,\left[ y \right]} }}_{Bd - MeHgInteraction} + \underbrace {{\left( {\beta_{{L_{s} }} + b_{{L_{j} }} } \right)L_{i,s,j,\left[ y \right]} }}_{SVLEffect} \hfill \\ \end{gathered}$$

which has the following indices: *i* = individual, *s* = species, *j* = population, *y* = calendar year, *x* = sex. The α_s_ and β_s_ coefficients designate species-specific fixed effects (intercept and slopes respectively), while the α_x_ coefficient describe a sex-specific intercept. The various a_j_ and b_j_ coefficients designate the population-specific conditional modes of the random effects (aka ‘deviates’) for the matching α_s_ and β_s_ covariates. Population-level random effect deviates were modeled as being drawn from a multivariate normal distribution with a mean of zero and covariance matrix to be estimated. See *Supplemental Methods: Models* for adjustments for the 10 populations with insufficient MeHg data.

To model the apparent survival of individual *i* (of species *s*) in population *j* between primary periods within a season we used the following simpler linear predictor:

2 $$\log it(\varphi_{(W)[i],s,j,[t]} ) = \alpha_{s} + a_{j}$$

where the index *t* is used to designate primary periods. Thus, within-season apparent survival was modeled using only a fixed effect for species (*s*) and random effect for population (*j*). The indices for individuals (*i*) and sampling occasion (*t*) are given brackets to indicate that across all within-season transitions between primary periods, each individual of a given species *s* in population *j* were constrained to have the same apparent survival.

Models for SVL, Bd infection load, and MeHg, our treatment of imperfect detection (including discussion about our treatment of sub-sites), and details on the intricacies of fitting the CJS model in *Stan* are described in detail in the Supplemental Methods.

### Model fitting

We fit our model in *Stan* 2.21.0^70^, interfaced through R 4.2.3^71^ using the package rstan^72^. We fit our primary model to 10 of the 20 populations: 9 populations in which at least 25% of individuals were measured for MeHg (Table S1) and inclusion of the Lily Pond population of boreal chorus frogs (14% of individuals measured for MeHg) to include both populations of boreal chorus frogs. During exploratory analysis this proved to be approximately the minimum percentage required to achieve model convergence. We obtained estimates for the other 10 populations using our secondary model that estimated MeHg as a population-level covariate and did not include the individual-informed Bd-MeHg interaction term in survival (See Supplemental Methods: Models).

Models were fit using 4 chains with random starting conditions and run until convergence (defined as an $\widehat{\text{R}}<1.01$ and an effective sample size greater than 1000 for all fixed effects and random effect variances; some individual random effect conditional modes did not meet this criterion). This took 4000 samples per chain with a thinning rate of 2 for both models. Both *Stan* models and all accompanying code to clean data and fit them are available at https://github.com/NEARMI/Bd_CMR/tree/Scientific_Reports. All priors for all parameters are clearly documented in the model text files in the section labeled “Priors”.

### Article impact statement

We quantify the impact of disease and a contaminant on multiple species and populations of amphibians across the United States.

## Supplementary Information

Supplementary Information.

## Data Availability

All data needed to evaluate the conclusions in the paper are present in the paper and/or the Supplementary Materials, and can be found at DOI: 10.5066/P9LSR4HY.
