# Supplementary material for: Independent and interactive effects of disease and methylmercury on demographic rates across multiple amphibian populations
Source: Sci Rep. 2025 May 19;15:17314. doi: 10.1038/s41598-025-99839-3 (PMC12089440; doi:10.1038/s41598-025-99839-3)
Supplement: Supplementary file 1 — Supplementary Information. [file 41598_2025_99839_MOESM1_ESM.pdf]

Supplementary Materials for

Independent and interactive effects of disease and methylmercury on demographic rates across multiple amphibian populations

This PDF file includes:

Supplementary Text

References

Figs. S1 to S17

Tables S1 to S4

Supplementary Text

Field Sampling

Animals were captured by hand, using dip nets, or using traps. Permits are listed in **Table S4**. Each animal was handled separately using either disposable, powderless vinyl gloves or new, clean plastic bags to avoid cross contamination. All captured animals were uniquely marked (or their mark recorded) and examined to determine sex; most animals were measured (snout-vent length [SVL] and mass). In many cases, individuals captured for the first time within a primary period were swabbed to quantify *Bd* load; some individuals were swabbed on repeat captures while other individuals were never swabbed (for further details on animal swab frequency see **Table S2**). A single tissue sample (single toe or tail clip) also was taken from individuals in 17 of our 20 sampled populations to assess MeHg concentrations (**Table S1**). After processing, animals were released at the location of capture.

To obtain Bd samples, rayon-tipped sterile swabs were rubbed over the plantar side of one front and one hind limb, the ventral tail surface (salamanders only), the dorsal side of the body, and the ventral surface of the body 5 times each. Each swab was placed individually into a sterile plastic vial with 20 µl of sterile DNA-ase free deionized water [1]. Swabs were chilled immediately after field collection in a cooler of ice packs and subsequently frozen at ≤-20ºC. Frozen swabs were sent to the U.S. Geological Survey's National Wildlife Health Center in Madison, Wisconsin, and stored frozen at -80ºC until analysis. All field methods were conducted under appropriate Scientific Collecting Permits (SCPs), Recovery Permits, and Institutional Animal Care and Use Protocols.

Many of our amphibian populations were sampled using two or more easily differentiable primary periods within each calendar year, with a large gap in time between the last primary

period in year *y* and first primary period in year *y + 1*. In general, most eastern newt and ranid frog populations were sampled similarly using this design (see **Figure S11**). For example, in the Oregon spotted frog population [Dilman Meadows], sampling occurred in two distinct primary periods each year (e.g., on August 15, 16, and 17 and on September 24, 25, and 26 in 2019). However, other populations, for example the Colorado boreal chorus frog populations [Matthews Pond and Lily Pond], were sampled using only a single primary period each year (**Figure S11**). The strongest outliers in terms of timing of sampling were the two frosted flatwoods salamander populations and one eastern newt population (all in Florida), which were sampled sparsely but semi-continuously during 8 and 10 months of the year, respectively (**Figure S11**). This sampling made it difficult, though still possible (**Figure S12**), to establish time gap thresholds (numbers of days between consecutive samples) with which to define primary periods, secondary periods, and between-year transitions.

Laboratory Methods

Analysis of Bd loads

We extracted Bd DNA from swabs as described by [2] except that 125 µl of PrepMan® Ultra Sample Preparation Reagent (Applied Biosystems, Foster City, CA) and 100 mg of zirconium/silica beads (Biospec Products, Bartlesville, OK) were used so that the entire swab was immersed. The bead-beating steps were conducted using a FastPrep®-24 homogenizer (MP Biomedicals, Santa Ana, CA). We used a real-time TaqMan PCR for duplex detection of Bd and *B. salamandrivorans* (Bsal) on the extracted DNA as described in [3]. We ran reactions on the 7500 fast real-time PCR system (Applied Biosystems, Foster City, CA) using QuantiFast Probe RT-PCR mastermix kit with ROX dye (Qiagen, Valencia, CA) and BSA as per the kit

instructions. We used 5 µL of the PrepMan® solution containing the extracted DNA as template for the PCR. We included a negative extraction control and a standard curve run in duplicate on each PCR plate. The standard curve consisted of five different concentrations of the target sequence for Bd and Bsal inserted into plasmids. The concentrations of the standards occurred at ten-fold dilutions ranging from 110–1,100,000 copies (0.5–5000 fg DNA) per reaction (on some initial runs, the standard range was 11–110,000 copies per reaction). The threshold for signal detection was set at 5% of the maximum fluorescence of the standards run for that assay. We calculated the efficiency of each run using standard curve amplification and repeated PCR plates with an efficiency of less than 90% or greater than 110%. We defined a Bd detection if a detectable signal with a logarithmic amplification curve existed at 40 or fewer PCR cycles and no detection in all other cases. We calculated the intensity of a detection based on a log-linear correlation of PCR cycle detected and standard curves of artificial standard of known target sequence copy number. We report all intensity data in units of estimated target copy number, as opposed to zoospore equivalents, because the number of PCR target ITS sequence copies varies by Bd lineage and the distribution of Bd genetic lineages is not well characterized across the U.S.A. [4].

Methylmercury analysis

Amphibian tissue clips were freeze dried for 48–96 hours until a constant mass was achieved. Tail clips with a dry mass > 50 mg were homogenized to a fine powder using stainless steel scissors and glass rods. All toe clips with a mass < 50 mg were analyzed whole. Methylmercury (MeHg) concentrations were measured on all toe and tail clips following EPA method 1630 [5].

Briefly, entire tissue clips (or approximately 10 mg of dried, homogenized sample) were digested in 4 mL of 30% nitric acid at 60 °C overnight (~15 hours) and then ethylated with 1% sodium tetra-ethylborate prior to being analyzed via cold-vapor atomic fluorescence spectrometry on a MERX-M (Brooks Rand Instruments, Seattle, Washington, USA) automated methylmercury analyzer. Digests of toe clip samples weighing < 1 mg were analyzed in triplicate and the average of the triplicate values was used for those samples. An exact mass for each sample was recorded prior to analysis and used to calculated MeHg concentration in the tissue clips. Quality assurance measures included analysis of two independently derived liquid calibration standards, two certified reference materials (either scallop tissue [IAEA-452; International Atomic Energy Agency, Vienna, Austria], DORM-4, or TORT-3), reagent blanks, a matrix spike, and sample duplicates with every run of 72 samples. Average (± standard error) percent recoveries were 100.0 ± 0.3% and 100.8 ± 0.2% for liquid standards (n = 579), and certified reference materials (n = 1276), respectively. Matrix spike recoveries for MeHg averaged 106.1 ± 0.6% (n = 276) and the mean relative percent difference for all duplicates was 4.0 ± 0.3% (n = 162). Method blanks for MeHg ranged from < 0.01 to 0.58 pg with a median of 0.03 pg (n = 986).

Models

A basic Cormack-Jolly-Seber model

For a given population, the fundamental data are:

- I: the total number of unique individuals ever captured
- T: the total number of capture periods
- $y_{i,t}$: a Boolean (0, 1) indicating if individual i was captured at time t

In its simplest form, the model has two primary parameters:

- ϕ_t: an estimate of 'apparent survival', the probability that an individual alive at time *t* remains alive in the population until time *t + 1*
- p_t: the probability that an individual alive at time *t* is captured at time *t*

In a classic *JAGS* [6] implementation, the model relies on a discrete latent (unobserved) parameter $z_{i,t}$, which indicates whether individual *i* is alive at time *t*:

$$z_{i,t} \sim \begin{cases} Bernoulli(\varphi_{t-1}) & z_{i,t-1} = 1 \\ 0 & z_{i,t-1} = 0 \end{cases} \qquad \textbf{Eq. S1}$$

Covariates can easily be included to allow φ and *p* to vary among individuals and across time. This is commonly done by modeling these parameters using a linear predictor on an unconstrained scale (-∞, ∞) that is transformed to the probability scale with a logit or probit transformation. In a single population, the survival probability of individual *i* from sampling occasion *t* to *t + 1* could be modeled, for example, as a function of an individual's size and environmental temperature. Random effects can also be included to help control for variation among repeated measures within groups (e.g., individuals from a site may vary in their average survival probability because of many unobserved characteristics).

Our models

Below we describe our modifications to the basic CJS model described above. Note that all sections, unless explicitly stated otherwise, apply to both CMR models (individual- and population-level MeHg).

Multiple populations. We estimated the effects of individual- and population-level covariates on individual survival (φ) and detection (p) using a combination of fixed and random effects. Because many species were only sampled in one location, we used 'population' (species-by-site) as the levels of each random effect. We use 'population' instead of 'site' for our random effect grouping to better capture species-unique responses to site characteristics. Details on how these fixed and random effects contribute to survival and detection are described in *"Robust design"* below.

Latent variables. Our model includes three individual-level continuous latent variables: SVL, Bd load, and MeHg concentration.

Individual SVL. Snout-to-vent length of individuals was measured inconsistently across many populations. Individuals were either: measured on every capture, measured on a subset of captures (e.g., either once per primary period or only on first overall capture), or never measured (e.g., eastern newts in MA were not measured in 2020 due to the disruption of standard field protocols because of the COVID-19 pandemic). For simplicity, we used a single average SVL for each individual for models of Bd, MeHg, and survival instead of fitting a growth model to estimate SVL over time. To obtain these average values for individuals with no missing SVL or for individuals with at least one SVL measurement, we simply averaged across all repeat measures for each individual. To estimate the SVL of individuals with no measures, we fit a linear model with sex and species as covariates using the SVL of all individuals with measures, and then drew values from the fitted model (within the CMR model to propagate uncertainty) for the individuals with no measures of SVL, using their sex and species as covariates. SVL

(measured and imputed) were centered and scaled (within species) and used as a predictor in the estimates for survival and detection.

Bd load. Relatively little is known quantitatively about the factors controlling within-individual variation in Bd load throughout a season; however, it is expected that Bd loads will show strong seasonality because of Bd's strong non-linear growth rate across temperature [7, 8]. In the Pennsylvania (PA) eastern newt population [Scotia Barrens] (we use square brackets for site names), for example, individual Bd loads roughly follow an inverted quadratic with a peak in early June (**Figure S13**), providing some support for this conjecture. Optimally, we would have used temporal sampling such as occurred in PA to model individual variation in Bd loads over time in all populations given that a population-specific covariate for `time' (i.e., Julian date) would help to separate individual from temporal variation, which is conflated when a `time' covariate is not included (i.e., if some individuals are captured only early in a season while other individuals are captured only mid-late season, individual-unique estimated Bd load deviates will be conflated with seasonal Bd dynamics). However, three sampling constraints limited our ability to model seasonal variation in Bd loads.

First, most populations were sampled over a narrow time period (often because of animal life-history constraints; for example, western toads are only available for detection for a short breeding season). A narrow window makes it impossible to model Bd growth either phenomenologically as a function of Julian day, for example, or mechanistically as a function of environmental temperature using Bd's thermal performance curve (e.g., see western toads: **Figure S14**). Viewed at a population level, Bd loads show a distinct lack of temporal variation over the narrow time period in which most populations were sampled (e.g., see *Rana* spp.:

Figure S15). Second, sampling in most populations began after a large proportion of animals were already infected in a given year (e.g., see **Figure S16**). Even sampling that was able to capture increasing prevalence (due to either increasing loads and/or new infections) in some years revealed substantial year-to-year variation (e.g., see [Blackrock Complex] in **Figure S16**) that was unobservable in most populations. Finally, relatively few individuals had multiple swabs taken in a single year with which to inform a continuous time within-individual Bd growth model (**Table S1**).

In one model iteration we explored using MeHg concentration to predict Bd loads, but removed MeHg as a predictor from our model given the lack of individuals with MeHg measures in most populations (**Table S1**) and nearly zero variation in Bd explained by MeHg (e.g., see **Figure S17**). Ultimately, we modeled individual-specific average Bd loads in each year using each individual's sex, species identity, and SVL, as well as average yearly site temperature and both a population-by-year random effect and an individual-level random effect. Multiple swabs of a given individual within a year were treated as repeat measures, which were important for informing the individual-level random effect (i.e., to separate individuals with generally high loads from those with generally low loads due to unobserved characteristics such as condition and immune system functioning). We treated multiple swabs as repeat measures because of a lack of sufficient temporal coverage in many populations to fit a seasonally dynamic model (see above and **Figures S12 and S15-S17**). Specifically, we modeled the log of individual *i*'s Bd load (of species *s,* sex *x*, population *j*, calendar year *y*, and swab occasion *o*) as:

$$\mathrm{Bd}_{i,s,x,j,y,[o]} = \underbrace{\alpha_s + \alpha_x + a_{j,y} + a_{i_j}}_{\text{Intercept}} + \underbrace{\beta_L \cdot L_{i,s,x,j,[y],[o]}}_{\text{SVL Effect}} + \underbrace{\beta_T \cdot T_{[i],[s],j,y,[o]}}_{\text{Temperature Effect}} + \varepsilon_{i,s,x,j,y,o} \qquad \textbf{Eq. S2}$$

where α_s and α_x are species-specific and sex-specific intercepts, respectively, and $a_{j,y}$ and a_{i_j} are population-by-year and individual-level random intercepts, respectively. Individual-level random

intercepts (a_{i_j}) were drawn from population-specific Normal distributions with means of 0 and variances to be estimated. The coefficient β_L gives the effect of individual SVL ($L_{i,s,j,[y],[o]}$) on Bd load, which has been used previously to explain variation in infection among individuals [9]. The coefficient β_T gives the effect of population-level yearly average temperature ($T_{[i],[s],j,y,[o]}$). We use brackets in **Eq. S2** and all other equations to notate indices over which an identical value was repeated. For example, we include [*i*] in temperature (T) to make it explicit that we are assuming all individuals were exposed to the same temperature (similarly, [*o*] to indicate the use of a yearly average site-level temperature across any repeat swabs within an individual). The residuals ($\varepsilon_{i,\, s,\, j,\, y,o}$) were assumed to be Normally distributed with an error variance to be estimated. Estimated individual yearly Bd loads were centered and scaled (across all populations) and used as a predictor in the estimates for survival and detection.

MeHg concentrations, populations with sufficient individual-level MeHg data. Individual-level MeHg concentrations were modeled using the following Gamma regression:

$$\mathrm{MeHg}_{i,s,j} \sim \Gamma\left(\psi, \omega_{i,s,j}\right)$$

$$\omega_{i,s,j} = \frac{\psi}{\mu_{i,s,j}} \qquad \textbf{Eq. S3}$$

$$\log(\mu_{i,s,j}) = \alpha_s + a_j + \beta_L \cdot L_{i,s,j} + \beta_D \cdot D_j$$

where individual-level MeHg concentrations ($\mathrm{MeHg}_{i,s,j}$) are modeled as Gamma distributed with a 'shape' ψ and 'inverse scale' ω. The expected value for each individual is given by $\mu_{i,s,j}$, which is modeled with a linear predictor on the log scale. In this linear predictor α_s and a_j are species-specific fixed intercepts and population-specific random effects, respectively; the subscripts *i* and *j* denote individual and population. Here $L_{i,s,j}$ and β_L give the SVL (data or imputed) of individual *i* of species *s* in population *j* and the regression coefficient for the effect of SVL on

MeHg, respectively. The coefficient β_D gives the effect of water drawdown (% of water disappeared), which can contribute to the methylation of mercury [10]. Drawdown was measured at the subsite level as categorical values between 1 and 4, which stood for binned continuous percentage values (1: 0-25%, 2: 26-50%; 3: 51-75%; 4: 76-100%). To obtain a continuous value for each population, we converted each subsite's categorical value to the center of the appropriate binned range and then took the average across subsites. The second line of this series of equations transforms the expected value to the 'shape' and 'inverse scale' parameters as used in the Gamma regression; see the *Stan* manual [11] for details on this transformation. MeHg concentrations (measured and imputed) were centered and scaled (across all species) and used as a predictor in the estimates for survival and detection.

MeHg concentrations, populations with insufficient individual-level MeHg data. In our secondary model fit to the 10 populations without sufficient individual-level MeHg measures, we estimated the average MeHg concentration in each population using all of the individuals with measured MeHg with the following Gamma regression:

$$\mathrm{MeHg}_{i,j} \sim \Gamma(\psi, \omega_{i,j})$$

$$\omega_{i,j} = \frac{\psi}{\mu_{i,j}} \qquad \textbf{Eq. S4}$$

$$\log(\mu_{[i],j}) = \alpha_s + a_j + \beta_D \cdot D_j$$

Because each measured individual's MeHg in population *p* informs just the population mean, and because the full linear predictor for $\mu_{[i],p}$ contains no individual-specific predictors, we were able to use the estimated coefficients in the linear predictor to estimate the mean MeHg concentration in population *j*. Population-specific MeHg values were then scaled and used as a predictor in the estimates for survival and detection.

Survival, populations with insufficient individual-level MeHg data. These estimated individual-level MeHg concentrations were then used in the estimates for between-season survival with:

$$\mathrm{logit}(\varphi_{(B)i,s,j,y}) = \underbrace{\alpha_s + \alpha_x + a_{i_j}}_{\text{Intercept}} + \underbrace{(\beta_{Bd_s} + b_{Bd_j}) \cdot Bd_{i,s,j,y}}_{\text{Bd Effect}} + \underbrace{\beta_{MeHg_s} \cdot MeHg_{[i],s,j,[y]}}_{\text{MeHg Effect}} + \underbrace{(\beta_{L_s} + b_{L_j}) \cdot L_{i,s,j,[y]}}_{\text{SVL Effect}} \quad \textbf{Eq. S5}$$

where the indices and coefficients have the same meanings as their main text counterpart (**Eq. 1**): *i* = individual; *s* = species; *j* = population; *y* = calendar year; *x* = sex; α_s and β_s = species-specific fixed effects; a_j and b_j = population-specific random effect deviates; L = SVL.

Detection. We modeled detection as:

$$\mathrm{logit}(p_{[i],s,j,t}) = \underbrace{\alpha_s + \alpha_e + a_j + a_{pp-ss-j}}_{\text{Intercept}} + \underbrace{\beta_{D_s} \cdot D_{[i],[s],j,[y]}}_{\text{Drawdown Effect}} + \underbrace{\beta_{V_s} \cdot V_{[i],[s],j,[y]}}_{\text{Vegetation Effect}} + \underbrace{(\beta_{L_s} + b_{L_j}) \cdot L_{i,s,j,[y]}}_{\text{SVL Effect}} \quad \textbf{Eq. S6}$$

which uses all of the indices as the equation for within-season survival, with one addition (*pp-ss-j*). The term $a_{pp\text{-}ss\text{-}j}$ describes the conditional modes of a random effect whose entries are defined by the unique combination of primary period (*pp*), collection of subsites sampled (*ss*), and population (*j*). We included variation among primary period to capture seasonal variation in detection probability. In short, this random effect sought to stand in for seasonal and subsite-level variation in detection driven by unmeasured covariates (further detail given below). Brackets around all indices apart from population (*j*) for drawdown and vegetation indicate that these are population-level covariates that did not vary by year. Here, to convert drawdown and vegetation (which were both recorded as binned values; 1: 0-25%, 2: 26-50%; 51-75%; 76-100%) to a continuous value for population *j* on occasion *t*, we converted each categorical binned

value to the center of the appropriate binned range and then took the average across all subsites sampled on occasion *t*.

To estimate the term $a_{pp\text{-}ss\text{-}j}$ we combined the captures from all sub-sites visited on each day a given population was sampled. The raw data clearly indicates that many animals were captured at many subsites over time; however, it is impossible to tell without an explicit latent movement model how much time animals spent in different subsites and which sub-sites individual animals favored. The term $a_{pp\text{-}ss\text{-}j}$ allows the overall daily detection probability to be dependent on which subsites were visited and thus seeks, in a way, to capture variation in animal subsite preferences.

This combination of between-season survival, within-season survival, and detection captures a highly simplified few of animal movement across a season. Specifically, we allow for no individual movement from, and return to, the population within a season (i.e., an animal that is not present to be detected for a time). Further, by modeling only apparent survival (death or permanent emigration) we cannot separate death due to Bd from more nuanced dynamics such as reduced or increased movement. For example, [12] found evidence for reduced movement by Bd-infected pool frogs (*Pelophylax lessonae*) in Sweden. If such a result holds in our populations, impacts of Bd on true survival could be biased downwards (less negative) because less infected individuals may leave the pond more readily than infected individuals. Such an explanation could, for example, help to explain positive median estimates between Bd and apparent survival.

Population sizes. Though not the focus of our model, we did estimate the size of population *j* of species *s* on sampling occasion *t* by summing three values: the total number of each sex caught on that occasion by the estimated probability of capturing that sex of that species on that day. We

note that this method is prone to giving noisy day-to-day estimates (due often to days when zero females or zero 'sex unknown' were captured), so we emphasize interpretation of the mean estimate within a year and broader trends over time as opposed to a single estimate on a specific day.

Stan. *Stan* uses Hamiltonian Monte Carlo (HMC) (specifically, a sampling method called the No-U-Turn Sampler [NUTS]) which requires a gradient calculation to generate step sizes between proposed samples. Importantly, this gradient information cannot be calculated on non-continuous functions, which are generated by discrete latent variables. Thus, models with discrete latent variables must be reformulated to be fit in *Stan*. In a model where a discrete latent variable is necessary, the strategy is to "marginalize over" (i.e., integrate out) the discrete latent variable. In brief, marginalizing over means writing the *joint distribution of the data and the parameters* (i.e., the numerator of Bayes' theorem—the posterior probability distribution of the parameters [including the latent discrete parameter] given the data) as a sum over all possible values of the discrete latent parameter.

In a CMR model, the parameters of interest φ and *p* (and all covariates that inform them) are fit using the complete capture history of each individual. Thus, it may appear that marginalizing over $z_{i,t}$ would therefore require a sum over *all possible capture histories for all individuals*. This would quickly become untenable given that the number of unique capture histories scales as 2^T where *T* is the number of sampling occasions. Fortunately, the problem can be constrained by considering three unique time periods for each individual: 1) all sampling occasions prior to an individual being captured for the first time; 2) the sampling occasions in-between an individual's first and last capture (for individuals captured only once this period is not relevant); 3) all

sampling occasions from the last time an individual is captured until the final time the population is sampled.

An individual does not contribute to the model likelihood until it is captured for the first time, so by definition entries of z can be ignored for each individual on all sampling occasions prior to each individual being captured for the first time (the first time period). For sampling occasions that fall into the second time period for a specific individual, the entries of z must equal one because we know that the individual was alive. This is true because of our assumption of a closed population. That is, our model does not consider temporary emigration—individuals that leave, are unavailable to be detected for a time, and then return; in our model formulation recaptured individuals are assumed to simply have gone undetected in sampling occasions between recaptures. Thus, from the very first time a population is sampled until the last occasion in which an individual was detected, survival and detection can be informed without the use of z. However, during the third time period (from the last occasion on which an individual was captured through to the final time the population was sampled), z plays the important role of separating a non-capture (a zero in the data) into one of two discrete underlying causes: 1) a living individual that was not detected ($z = 1$ but $y = 0$), or 2) a dead individual ($z = 0$ and $y = 0$). The solution for removing the model's reliance on z during this period is to calculate a quantity, referred to as χ_t, that is a function of φ and p and gives the probability that an individual, alive at time t, *will never be captured again*. By allowing χ_t to inform the likelihood during the third of the three time periods, the complete capture history gets to inform φ and p and the need for z has been removed. We provide an example calculation for χ_t here and then define how χ_t contributes to model likelihood.

In a population sampled twice, χ is easy to define. For an individual with capture history [1, 0], for example, the individual is not captured on occasion 2 with probability $(1 - \varphi_1) + \varphi_1 \cdot (1 - p_2)$. The first quantity $(1 - \varphi_1)$ gives the probability that the animal will die between sampling occasions 1 and 2 and the second quantity $(\varphi_1 \cdot (1 - p_2))$ gives the probability that the animal lives but is not captured during occasion 2. For an individual with a capture history of [1, 0, 0] in a population sampled three times, the probability that the individual will not be captured again after the first occasion (χ_1) is given by:

$$\chi_1 = (1 - \varphi_1) + (\varphi_1 \cdot (1 - p_2)) \cdot ((1 - \varphi_2) + (\varphi_2 \cdot (1 - p_3)) \qquad \textbf{Eq. S7}$$

Though this quantity is already starting to look unwieldy, it also makes logical sense. What it says is that the probability that an individual will not be captured on occasions two and three is equal to the probability that it dies between occasion one and two plus the probability that it lives to occasion two, *but does not subsequently get captured*, which is just χ_2. Thus, χ_t is in fact a recursive calculation given by:

$$\chi_t = (1 - \varphi_t) + \varphi_t \cdot (1 - p_{t+1})) \cdot \chi_{t+1} \qquad \textbf{Eq. S8}$$

That is, χ_t for any number of sampling occasions can be calculated by recursively working backwards in time starting with χ for the last transition. In *Stan* this recursive calculation can be written as a user defined function inside of the 'functions' block of the model (see [11] for more information).

Ultimately, inference is drawn on all parameters with three sampling statements that define the likelihood of the whole model, one each for survival (φ) and detection (p), and one for χ. Survival and detection (and thus all parameters that are a part of their linear predictors) are informed directly during the period in which we know that an individual was alive (the second of the three time periods defined previously). Recall that leading 0s before the first 1 in the capture

history of individuals (the first of the three time periods) do not inform the model likelihood and thus do not inform any parameter estimates. For all transitions in the second period the following sampling statement is used:

$$1 \sim Bernoulli(\varphi_{i,t})$$ **Eq. S9**

because we know that an individual lived through all transitions between the first occasion that it was captured and the transition defined by the occasion directly preceding the last time it was captured and the last occasion on which it was captured. That is, the one to the left of the tilde represents our certain knowledge (given our model formulation) that the animal lived through a given transition (from occasion t to occasion $t + 1$).

Detection is informed in the second time period (after we know that an individual is alive), through to the last time they are captured. That is, the first time p is informed by the data y is on the first occasion after an individual is captured, with:

$$1 \sim Bernoulli(p_{i,t})$$ **Eq. S10**

which means that the occasions spanned by **Eq. 9** and **10** are slightly different, as can be seen in the ‘Model’ block of the *Stan* model available at [*Link available upon acceptance*].

On the last occasion t that each individual i was captured, χ is used to inform the likelihood (and both φ and p as well given that it is a function of these quantities) with:

$$1 \sim Bernoulli(\chi_{i,t})$$ **Eq. S11**

Here, the one to the left of the tilde reflects that the outcome that actually occurred is that the animal was never captured again after occasion t (the definition of χ_t) (i.e., in the data for individual i after occasion t is all zeros).

As an illustrative example to show how different individuals inform the model likelihood and thus φ and p, consider three individuals with different capture histories in a population

sampled five times: one individual with a capture history [0, 1, 0, 1, 0], a second individual with a capture history [0, 1, 0, 0, 0], and a third with capture history [0, 0, 0, 0, 1].

Individual 1: In the first time period (just the first sampling occasion) individual 1 does not contribute because it is unknown if it was part of the population. There are two transitions over which we know the individual survived, the transitions between sampling occasions 2-3 and 3-4. Thus, the sampling statements used for this individual include φ_2 and φ_3 (**Eq. S9**) as well as p_3 and p_4 (**Eq. S10**). Finally, φ and p are further informed through $1 \sim \text{Bernoulli}(\chi_4)$ (**Eq. S11**).

Individual 2: Because individual 2 was only ever captured one time, individual 2 contributes to the model likelihood only through χ_2, the full calculation of which is shown in **Eq. S8**.

Individual 3: Because individual 3 was only ever captured on the very last occasion, they cannot contribute to the likelihood at all; φ estimates the probability of surviving to the next period (for which we don't have data), p cannot informed because we only learned of this individual's existence and then stopped sampling, and χ on the last sampling occasion is equal to one by definition (if we never sample again the probability of never capturing individual i again is ensured). For convenience we simply remove individuals that were only ever captured on the last sampling occasion (see ***Model fitting*** below).

Model Fitting. To fit both models we made the following data simplifications:

1. Combining the sampling of all subsites within each site, apart for when modeling daily detection probably with the use of the parameter $a_{pp\text{-}ss\text{-}j}$ as described in **Eq. S6**.

2. Removing any individuals that were only ever captured on the very last day that their population was sampled—because the CJS models are conditioned on captured individuals, those marked in the last period do not contribute to the model likelihood.
3. Collapsing the Bd swab results for 132 individuals that were swabbed two or more times on the same day. For these few data points three scenarios were possible: negative-positive, negative-negative, positive-positive. For the positive-positive records (49/132) the average of the two Bd load values was used, for the negative-negative records (47/132) the animal was taken to be negative for infection, while for the negative-positive records (36/132), the load in the positive sample was used.
4. Collapsing the 6 *Rana* spp. into their own genus-level category to inform one level of each species-specific fixed effect because 5 of these 6 species were only sampled at one site (two for the Columbia spotted frog), in many cases with a small sample size (**Table S3**).

Caveats

Extended details on study/model limitations.

Treatment of Bd

We point out that a single measure of `uninfected' or 'infected' (as opposed to a quantitative measure of load) also provides little bearing on whether that individual will have been infected at some point in the year, for how much of the year that individual was in infected, or how intense their infection was. Further, collapsing all loads (which may be quite variable in the population) to 'infected' forces the model to estimate a single mean effect, which could be difficult to detect if the effect of Bd is only large when Bd loads are high.

Another major issue with our Bd data was the vast variation seen among individuals (e.g., see **Figures S6, S14-S17**) and the large proportion of captured individuals never measured for

their Bd load (**Table S1**). Data exploration and/or model fits revealed extremely low explainable variance with any measured covariates (e.g., even length and sex which were included in the model). This made predictions for the Bd loads of unmeasured individuals highly uncertain, meaning that those individuals could contribute little to resolving the impact of Bd. While modeling Bd in these individuals could conceivably help some, the low explainable variation made it such that the sample sizes in each population roughly matched the number of individuals measured for Bd, not the number of individuals captured (causing a large loss in sample size in populations such as those of the boreal chorus frog and frosted flatwoods salamander, hence the wide CI for these species for the effects of Bd on survival, **Figure 2**). Future field and lab work is needed to explain this vast variation in observed Bd loads among individuals and to reduce the observation uncertainty in Bd load (which we ignored in this study) to pin down precise estimates of the effect of Bd.

Temperature

First, because we were unable to model continuous patterns of Bd throughout the year, it was difficult to use continuous values of temperature in a meaningful way. Thus, we simply used average temperature by year by population to help estimate average Bd loads seen in that population in that year. Second, the 1 km resolution for temperature we use in our model for Bd loads likely poorly reflects the temperatures that individuals are exposed to. Many factors influence the temperature that each individual is exposed to, including large variation across individual ponds or wetlands because of differences in tree cover, aquatic vegetation, and water depth. Further, the actual temperature that each individual experiences will vary due to microhabitat choice and basking behaviors. The temperature data we used roughly splits the

difference between the first two scales. While we do have measures of water depth, canopy cover, or both, some initial data exploration and model fitting revealed they had close to zero predictive power in explaining the variation in Bd loads among populations, thus we opted against further complicating the model with additional covariates. Finally, we use temperature without a lag in our model for Bd loads, which arguably does not well represent the dependence of Bd loads on environmental temperature. However, without a model for dynamics and because temperature in our model cannot help to explain the variation among individuals (i.e., we had no temperature measures at individual capture locations), we felt that a pursuit of exploring alternative lags was not a good use of resources.

Supplement References

[1] McKenzie J, Price SJ, Fleckenstein JL, Drayer AN, Connette GM, Bohuski E, Lorch JM. 2019. Field diagnostics and seasonality of Ophidiomyces ophiodiicola in wild snake populations. EcoHealth 16:141–150.

[2] Boyle AHD, Olsen V, Boyle D, Berger L, Obendorf D, Dalton A, Kriger K, Hero M, Hines H, Phillott R. 2007. Diagnostic assays and sampling protocols for the detection of Batrachochytrium dendrobatidis. Diseases of aquatic organisms 73:175-192.

[3] Blooi M, Pasmans F, Longcore JE, Spitzen-Van Der Sluijs A, Vercammen F, Martel A. 2013. Duplex real-time PCR for rapid simultaneous detection of Batrachochytrium dendrobatidis and Batrachochytrium salamandrivorans in amphibian samples. Journal of clinical microbiology 51:4173-4177.

[4] Rebollar EA, Woodhams DC, LaBumbard B, Kielgast J, Harris RN. 2017. Prevalence and pathogen load estimates for the fungus Batrachochytrium dendrobatidis are impacted by ITS DNA copy number variation. Diseases of Aquatic Organisms 123:213-226.

[5] USEPA. 1998. Method 1630: Methylmercury in water by distillation, aqueous ethylation, purge and trap, and cold vapor atomic fluorescence spectrometry. Washington, DC.

[6] Plummer, M. 2003. JAGS: A program for analysis of Bayesian graphical models using Gibbs sampling. In Proceedings of the 3rd international workshop on distributed statistical computing (Vol. 124, No. 125.10, pp. 1-10).

[7] Stevenson LA, Roznik EA, Greenspan SE, Alford RA, Pike DA. 2020. Host thermoregulatory constraints predict growth of an amphibian chytrid pathogen (Batrachochytrium dendrobatidis). Journal of Thermal Biology 87:102472.

[8] Turner A, Wassens S, Heard G, Peters A. 2021. Temperature as a driver of the pathogenicity and virulence of amphibian chytrid fungus Batrachochytrium dendrobatidis: A systematic review. The Journal of Wildlife Diseases 57:477-494.

[9] Grear D, et al. *In Press*. Pan-Amphibia distribution of the fungal parasite Batrachochytrium dendrobatidis varies with species and temperature. Ecological Monographs.

[10] Obrist D, Kirk JL, Zhang L, Sunderland EM, Jiskra M, Selin NE. 2018. A review of global environmental mercury processes in response to human and natural perturbations: Changes of emissions, climate, and land use. Ambio 47:116-140.

[11] Stan Development Team. 2022. Stan modeling language users guide and reference manual, 2.21.0. https://mc-stan.org.

[12] Sainsbury A, Yu-Mei R, Ågren E, Vaughan-Higgins R, Mcgill I, Molenaar F, Peniche G, Foster J. 2017. Disease risk analysis and post-release health surveillance for a reintroduction programme: the pool frog Pelophylax lessonae. Transboundary and emerging diseases 64:1530-1548.

Supplemental Figures

Figure S1. Estimates for the survival of the 'average' individual are more precise the larger the number of average captures per individual in the population (top). Estimates for the effects of Bd load on survival become more precise with increasing numbers of captures and swabs per individual in the population (bottom). Species included frosted flatwoods salamander (*Ambystoma cingulatum*), western toad (*Anaxyrus boreas*), eastern newt (*Notophthalmus viridescens*), boreal chorus frog (*Pseudacris maculata*), and six ranid frog species (*Rana* spp.). The six ranid frog species (and site location names) were: Columbia spotted frog (*Rana luteiventris*), Oregon spotted frog (*R. pretiosa*), foothill yellow-legged frog (*R. boylii*), California red-legged frog (*R. draytonii*), Sierra Nevada yellow-legged frog (*R. sierrae*), and Cascades frog (*R. cascadae*).

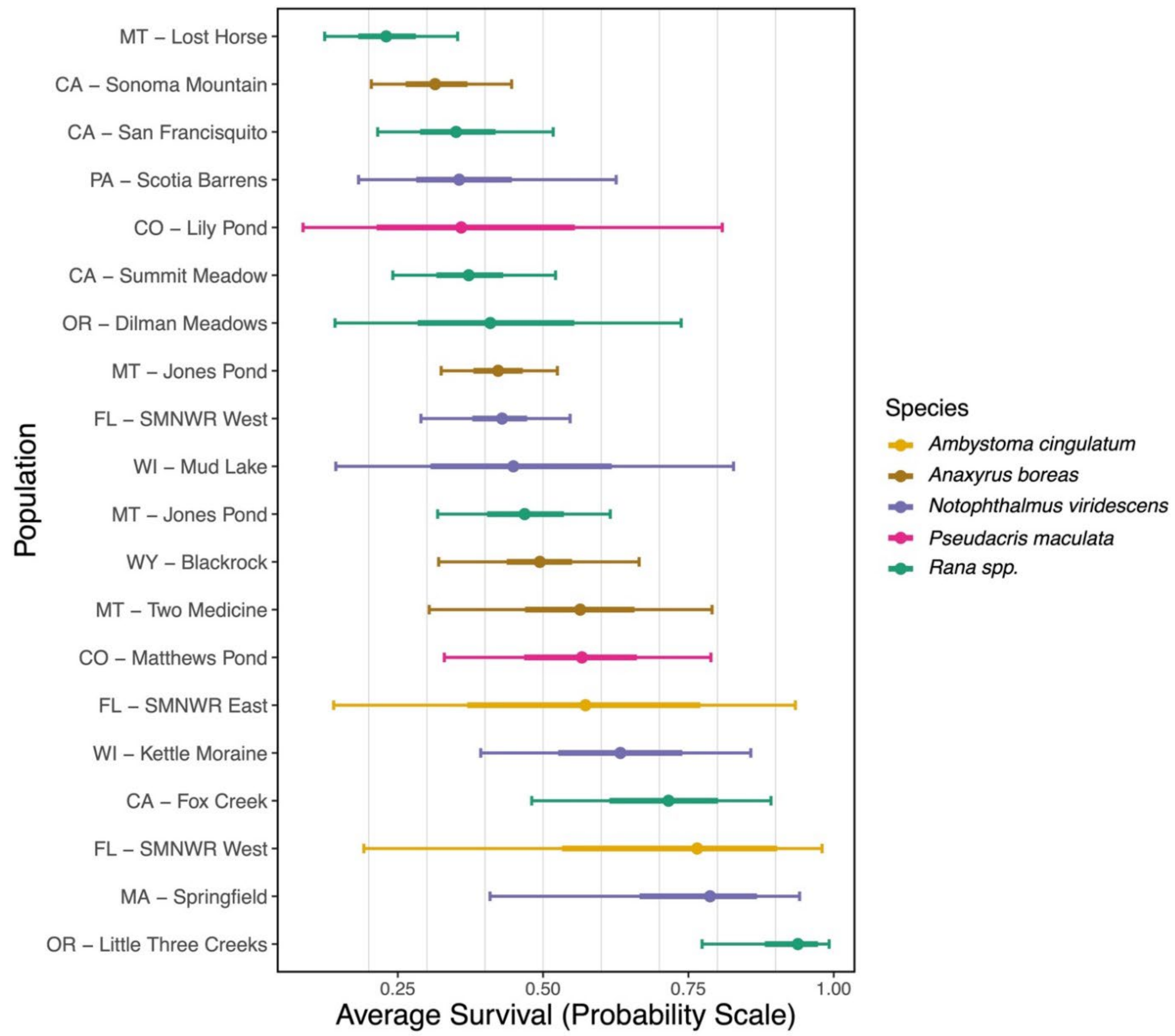

Figure S2. Yearly apparent survival estimates (the probability of remaining in the population between years) of an `average' individual (i.e., those with a mean yearly Bd load, SVL, and MeHg concentration) in all populations. Populations are sorted from top to bottom by median estimate (points). The wide intervals give 95% CI while the internal (thick) lines show 60% CI. Median estimates are as follows: OR - Little Three Creeks: 0.938; MA - Springfield: 0.787; FL - SMNWR West: 0.765; CA - Fox Creek: 0.716; WI - Kettle Moraine: 0.633; FL - SMNWR East: 0.573; CO - Matthews Pond: 0.567; MT - Two Medicine: 0.564; WY - Blackrock: 0.494;MT - Jones Pond: 0.468; WI - Mud Lake: 0.449; FL - SMNWR West: 0.429; MT - Jones Pond: 0.423; OR - Dilman Meadows: 0.409; CA - Summit Meadow: 0.372; CO - Lily Pond: 0.359; PA - Scotia Barrens: 0.356; CA - San Francisquito: 0.350; CA - Sonoma Mountain: 0.314; MT - Lost Horse: 0.230. Species included frosted flatwoods salamander (*Ambystoma cingulatum*), western toad (*Anaxyrus boreas*), eastern newt (*Notophthalmus viridescens*), boreal chorus frog *(Pseudacris maculata*), and six ranid frog species (*Rana* spp.). The six ranid frog species (and site location names) were: Columbia spotted frog (*Rana luteiventris*; Jones Pond, Lost Horse), Oregon spotted frog (*R. pretiosa*; Dilman Meadows), foothill yellow-legged frog (*R. boylii*; Fox Creek), California red-legged frog (*R. draytonii*; San Francisquito Creek), Sierra Nevada yellow-legged frog (*R. sierrae*; Summit Meadow), and Cascades frog (*R. cascadae*; Little Three Creeks).

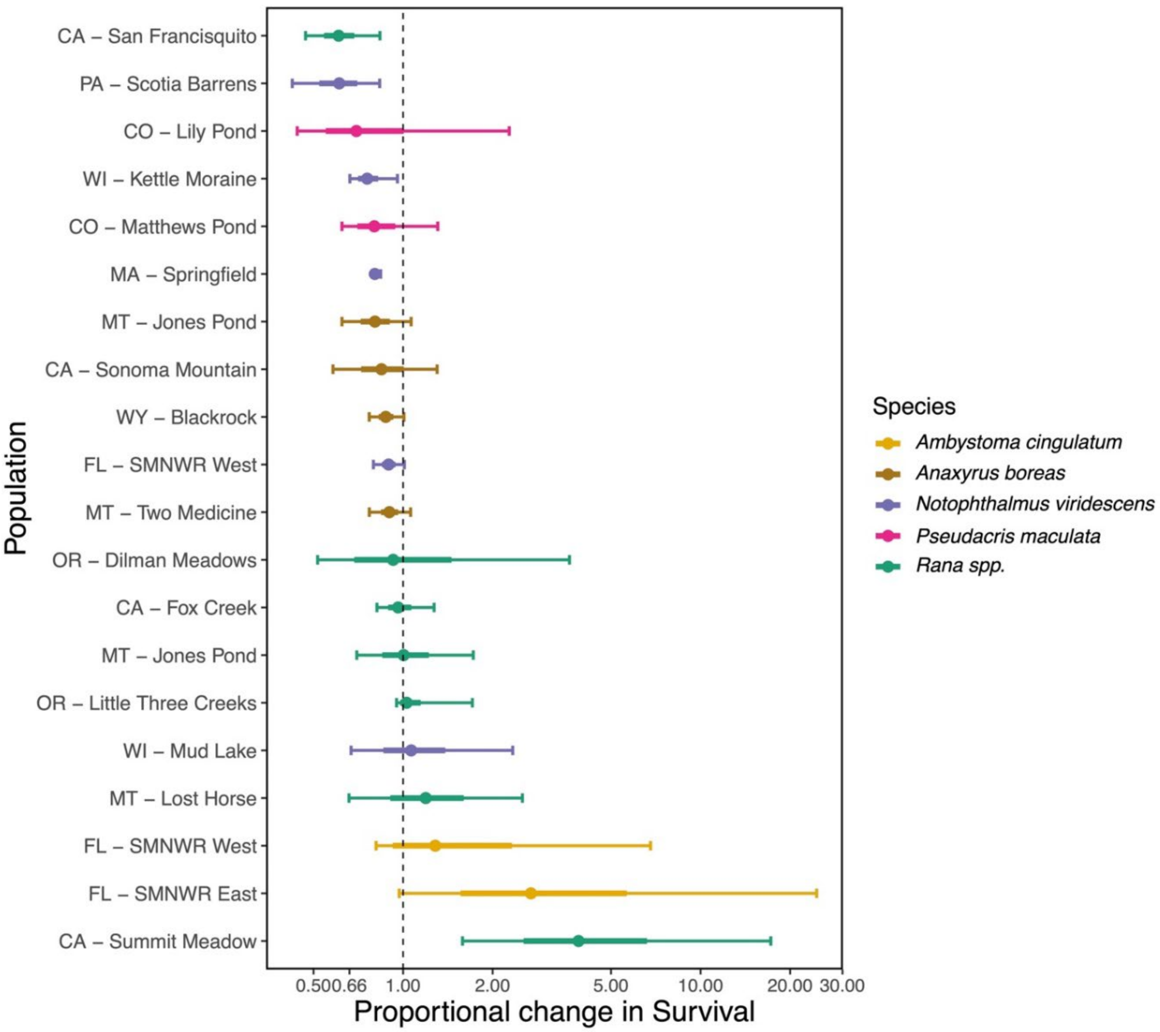

Figure S3. Estimated proportional increase (values greater than one) or decrease (values less than one) in survival probability with a one SD increase in Bd load from the mean Bd load. Uncertainty in mean survival and the effects of Bd are included in these CI; wide CI in the bottom few populations include highly unlikely values because of high uncertainty in these populations due to data sparsity. Points show medians of posterior distributions, thick uncertainty bands show 60% CI, and thin bands show 95% CI. Populations are sorted from largest negative to largest positive median estimate. Median estimates are as follows: CA - Summit Meadow: 3.89; FL - SMNWR East: 2.69; FL - SMNWR West: 1.28; MT - Lost Horse: 1.19; WI - Mud Lake: 1.06; OR - Little Three Creeks: 1.03; MT - Jones Pond: 1.00; CA - Fox Creek: 0.962; OR - Dilman Meadows: 0.926; MT - Two Medicine: 0.899; FL - SMNWR West: 0.894; WY - Blackrock: 0.874; CA - Sonoma Mountain: 0.846; MT - Jones Pond: 0.804; MA - Springfield: 0.803; CO - Matthews Pond: 0.800; WI - Kettle Moraine: 0.757; CO - Lily Pond: 0.696; PA - Scotia Barrens: 0.610; CA - San Francisquito: 0.607.

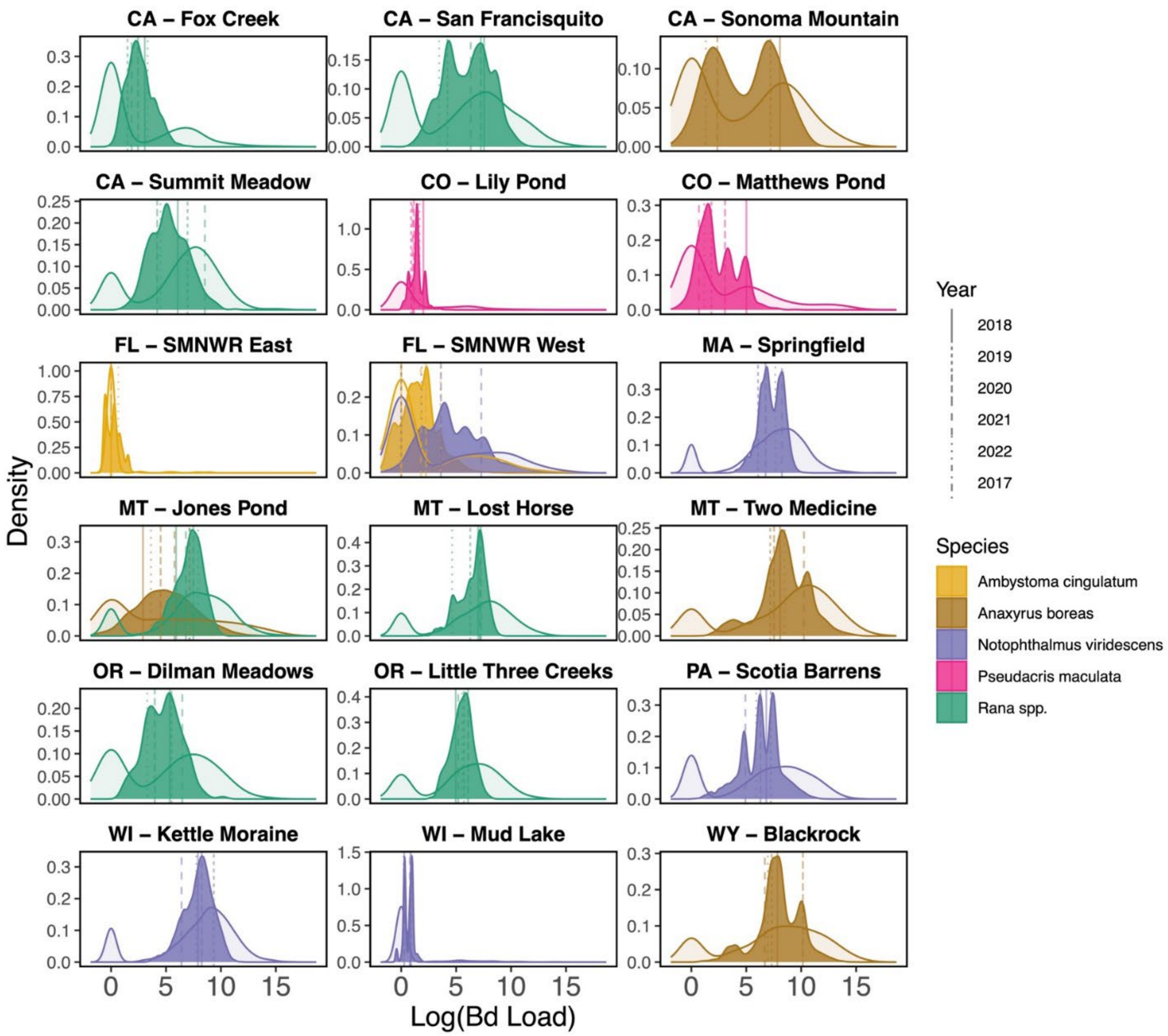

Figure S4. Distributions of estimated (opaque) and raw (translucent) average yearly Bd loads among all individuals within each population. Densities represent individual -by- year values, which results in multimodal predictive distributions in many populations; vertical lines show the variation in averages among years in the *raw data*. This figure highlights two important aspects of our data and modeling choices. Namely, because most individuals were only measured a single time for Bd load (**Table S3**) and sampling did not occur over much of the year in most populations (**Figure S1**), any individual data point contains relatively little information about that individual's load within the year. An important outcome of the random effects structure is that it pulls estimated individual yearly average values to the average estimated species -by- population -by- year value, which results in narrower estimated *average* Bd load distributions (opaque) than *raw individual value* Bd load distributions (translucent). In other words, individuals measured once and found to be uninfected were estimated to have a non-zero average load in the year (i.e., were infected at some point in the year). Similarly, individuals with very high sampled loads are pulled downwards (i.e., had lower values in other parts of the year). In the random effects framework, individuals with more samples are affected less by this attraction to the mean (i.e., individuals detected to be negative three times would be estimated to have a very low, but non-zero, average load).

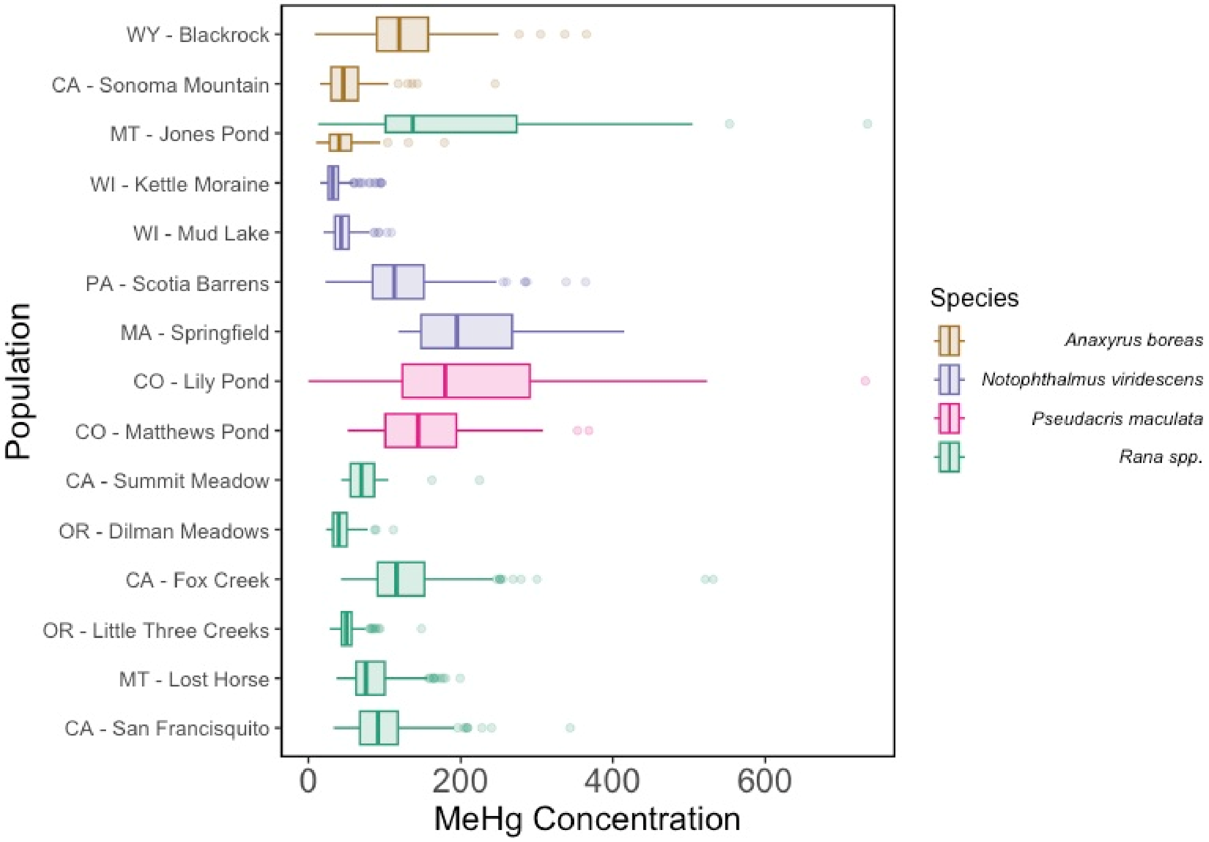

Figure S5. Boxplots showing MeHg concentrations (raw data) among individuals in the 16 populations in which MeHg was measured; four populations are not pictured because of zero MeHg measurements (FL - SMNWR East, FL - SMNWR West, MT - Two Medicine, FL - SMNWR West). **Table S1** lists, in all populations, the proportions of all captured individuals that were measured for MeHg.

Figure S6. Between-season survival of individuals in all 20 populations depending on their length (SVL) (scaled, x-axis). Points show medians of posterior distributions, thick uncertainty bands show 60% CI, and thin bands show 95% CI. In most populations being one SD larger (or smaller) than the average individual has a much larger effect on survival than either Bd or MeHg.

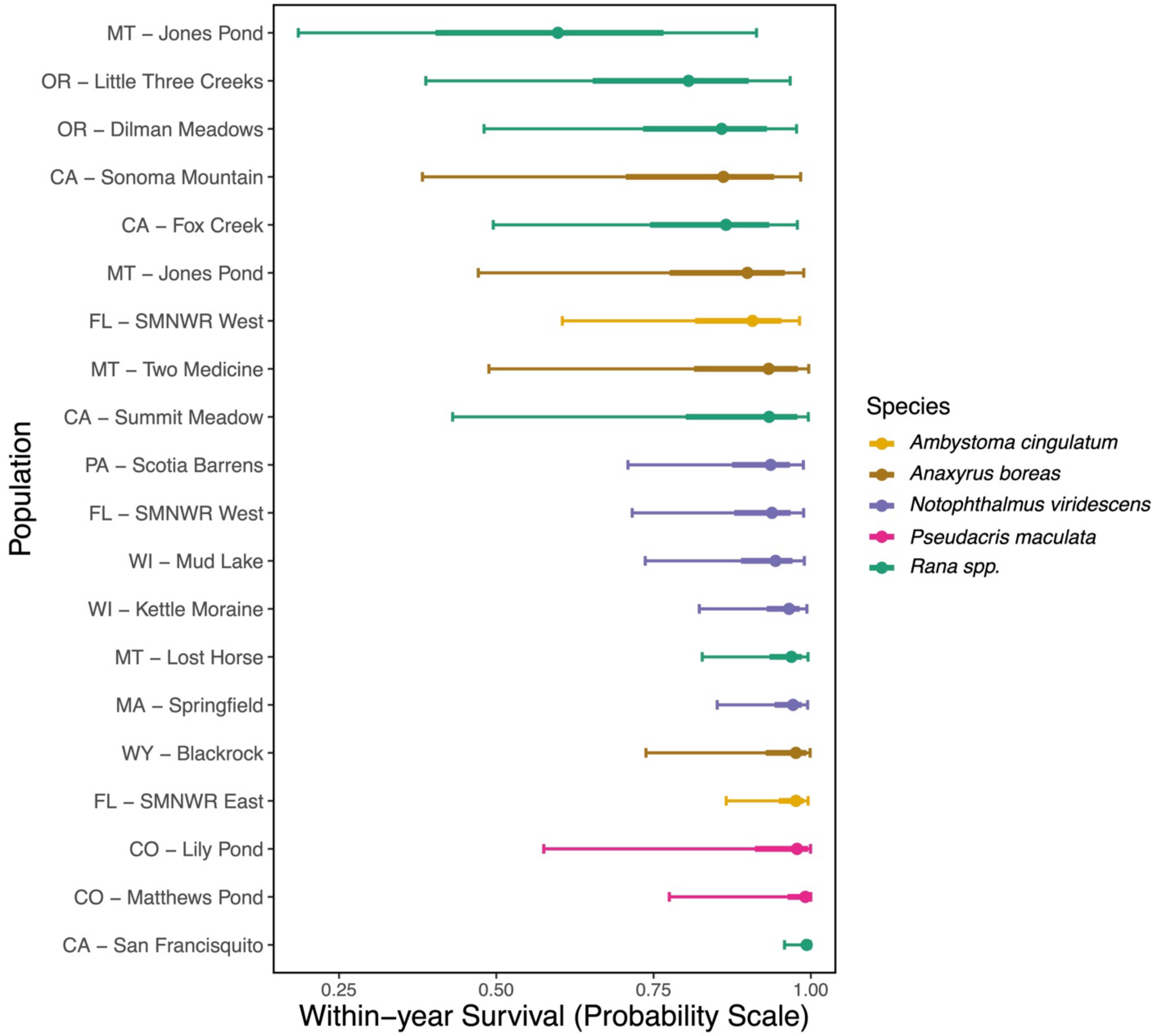

Figure S7. Species-level coefficient estimates for within-year apparent survival (probability of remaining within a population between primary periods). Points show medians of posterior distributions, thick uncertainty bands show 60% CI, and thin bands show 95% CI. In general, *Rana* spp. have the lowest median estimates and wide CI because of large time gaps between primary periods or only single primary periods within years. Median estimates are as follows: CA - San Francisquito: 0.99; CO - Matthews Pond: 0.991; CO - Lily Pond: 0.978; FL - SMNWR East: 0.976; WY - Blackrock: 0.976; MA - Springfield: 0.972; MT - Lost Horse: 0.969; WI - Kettle Moraine: 0.965; WI - Mud Lake: 0.944; FL - SMNWR West: 0.938; PA - Scotia Barrens: 0.936; CA - Summit Meadow: 0.934; MT - Two Medicine: 0.933; FL - SMNWR West: 0.907; MT - Jones Pond: 0.899; CA - Fox Creek: 0.865; CA - Sonoma Mountain: 0.861; OR - Dilman Meadows: 0.858; OR - Little Three Creeks: 0.806; MT - Jones Pond: 0.598.

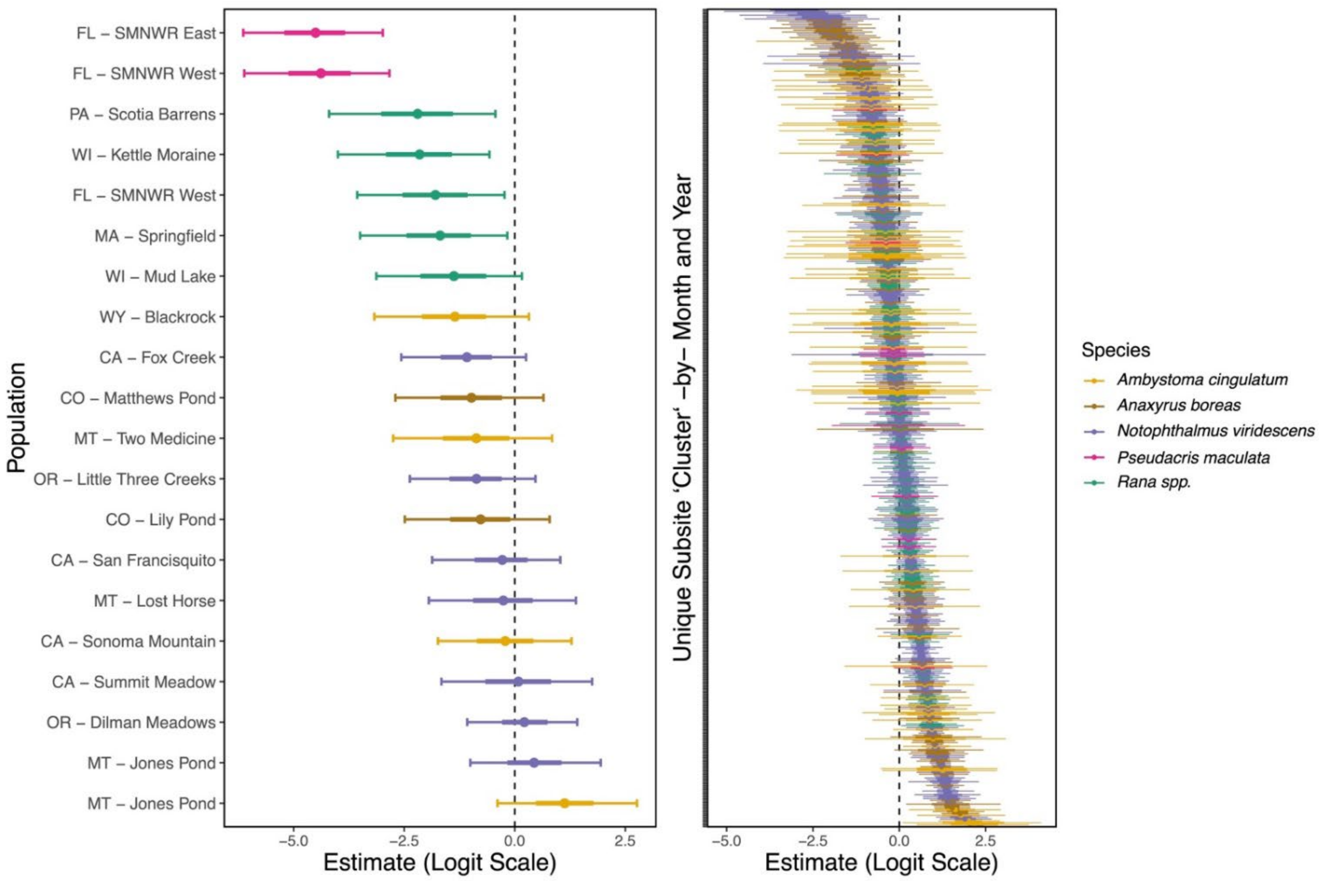

Figure S8. Random effect deviates (conditional modes) for detection, which affect the *per-sampling-event probability of capturing any specific individual,* with negative values decreasing detection probability. Population-specific detection random deviates are shown in the *left panel*. In the model, unique combinations of subsites sampled in a given month in a given year received their own random effect deviate; these are shown in the *right panel*. For example, in population 1, subsite A and B sampled in October, 2020, would be one level of this random effect. An additional trip within the same month and year to those same sites would be equivalent. This captured variation among subsites, seasonality, and year; i.e., regardless of species, individuals are particularly easy to find at times and particularly hard to find at other times. The conditional models of both random effects are sorted from top to bottom by median. The right panel is meant to illustrate the total variation in detection; specific deviates are not meant to be recovered from this figure. Points show medians of posterior distributions, thick uncertainty bands show 60% CI, and thin bands show 95% CI.

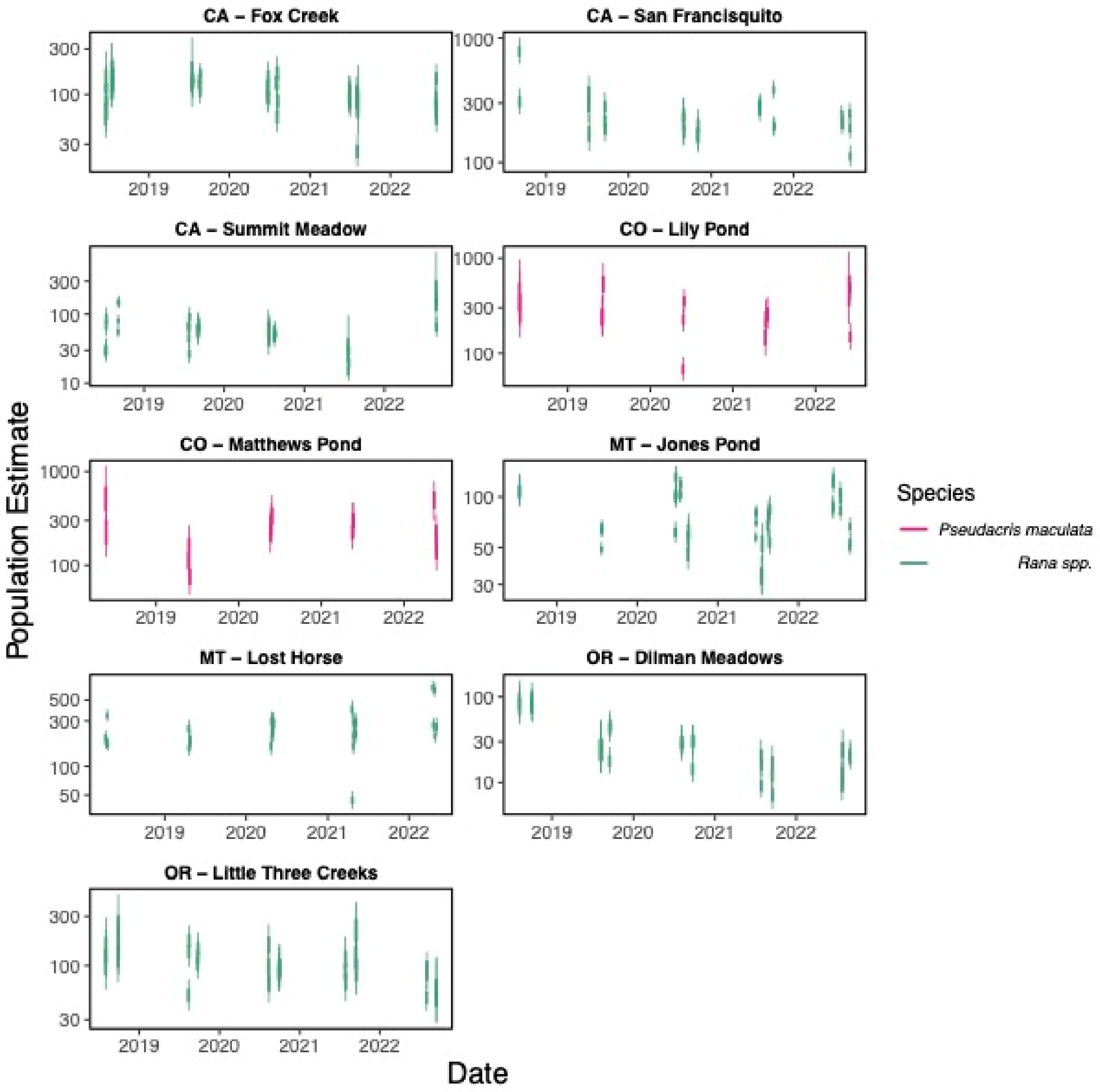

Figure S9. Population size estimates over time in all *Pseudacris maculata* and *Rana* spp. populations. Points show medians of posterior distributions, thick uncertainty bands show 60% CI, and thin bands show 95% CI.

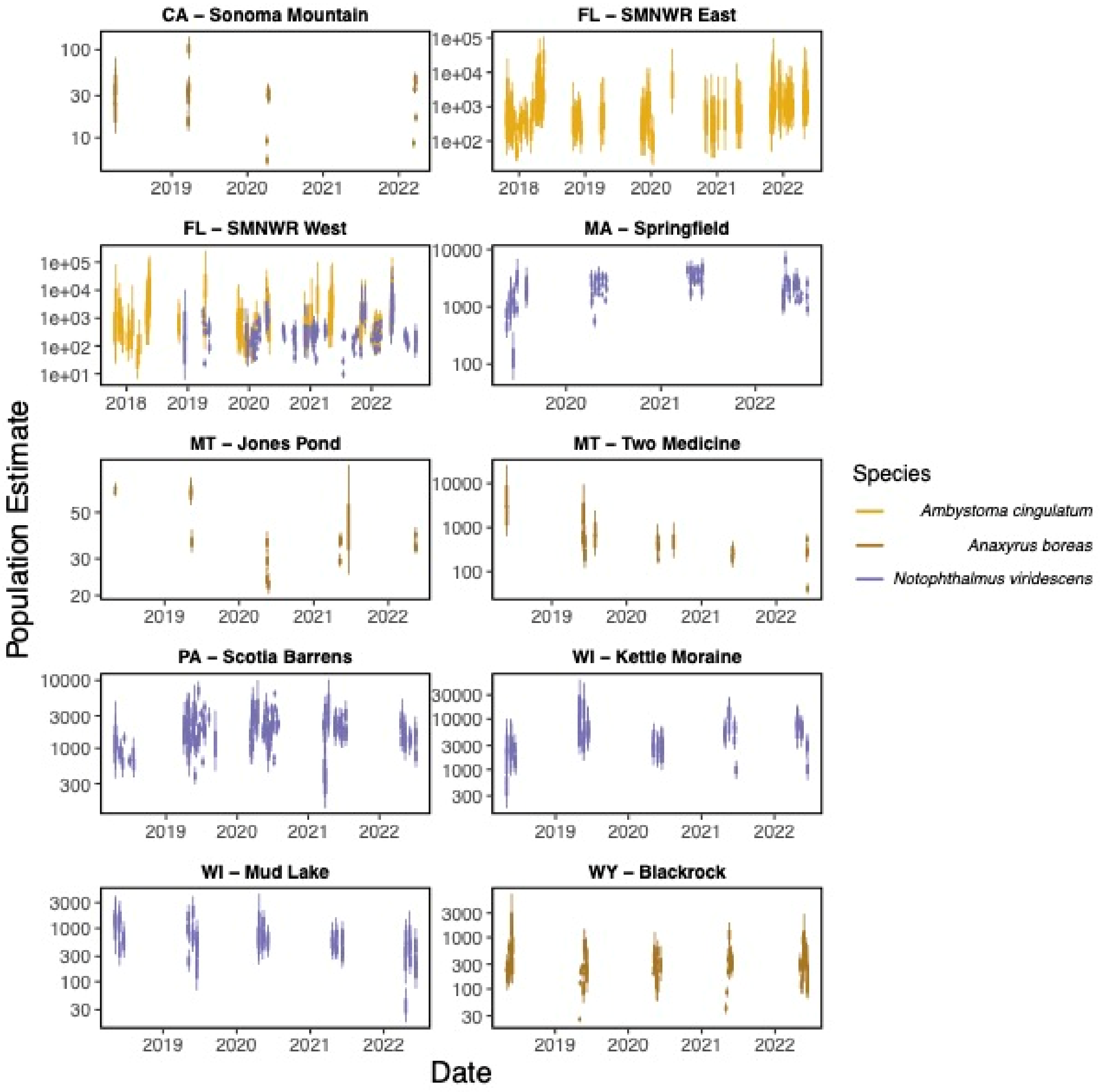

Figure S10. Population size estimates over time in all *Ambystoma cingulatum, Anaxyrus boreas,* and *Notophthalmus viridescens* populations. Points show medians of posterior distributions, thick uncertainty bands show 60% CI, and thin bands show 95% CI.

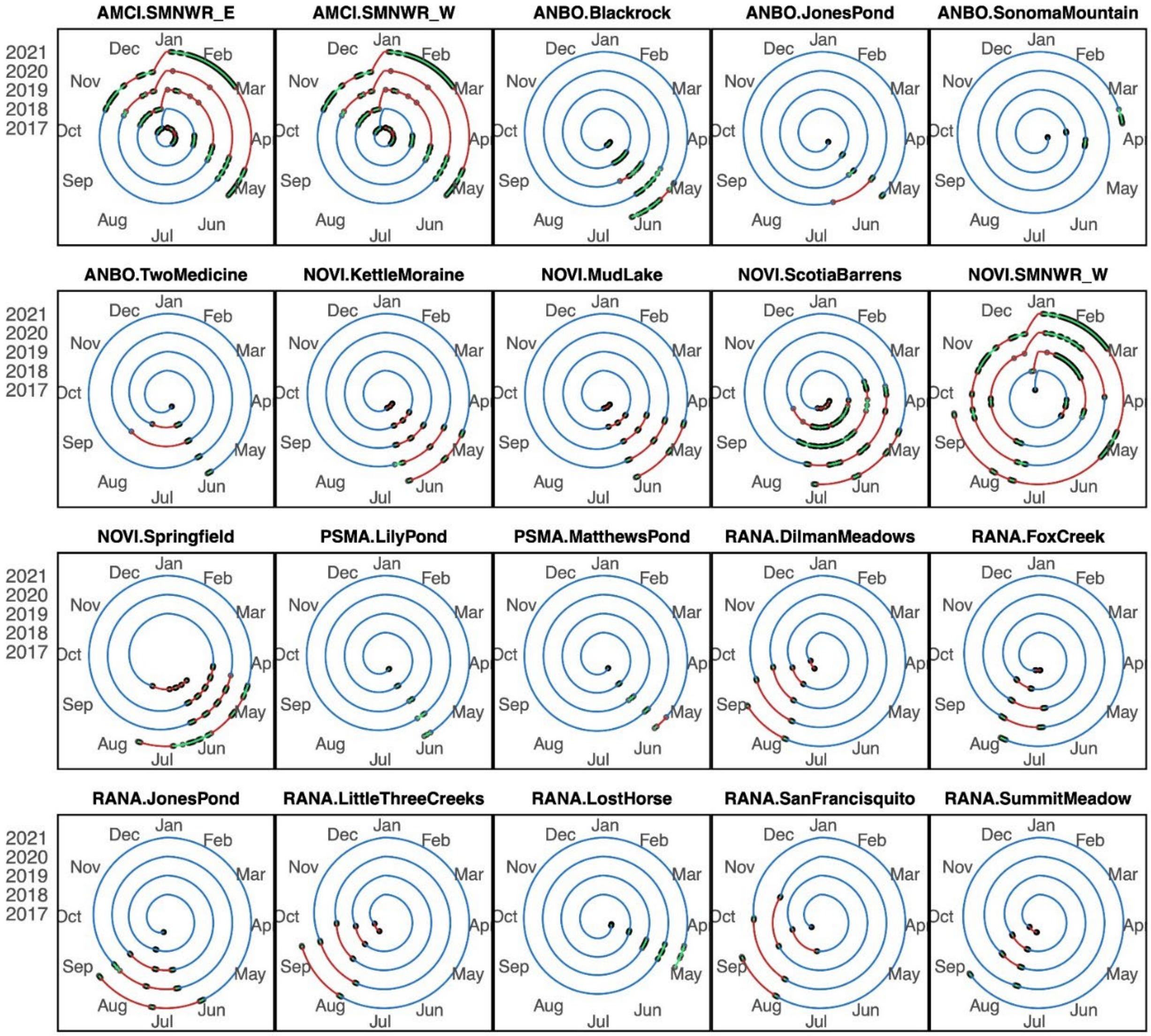

Figure S11. Sampling of all 20 populations. Points give dates of sampling events, moving through time (across years) from the inside outward. Blue lines connecting sampling occasions show time gaps given the ``offseason" designation; red lines show time gaps given the "onseason" designation; green lines show time gaps given the "closed population" assumption.

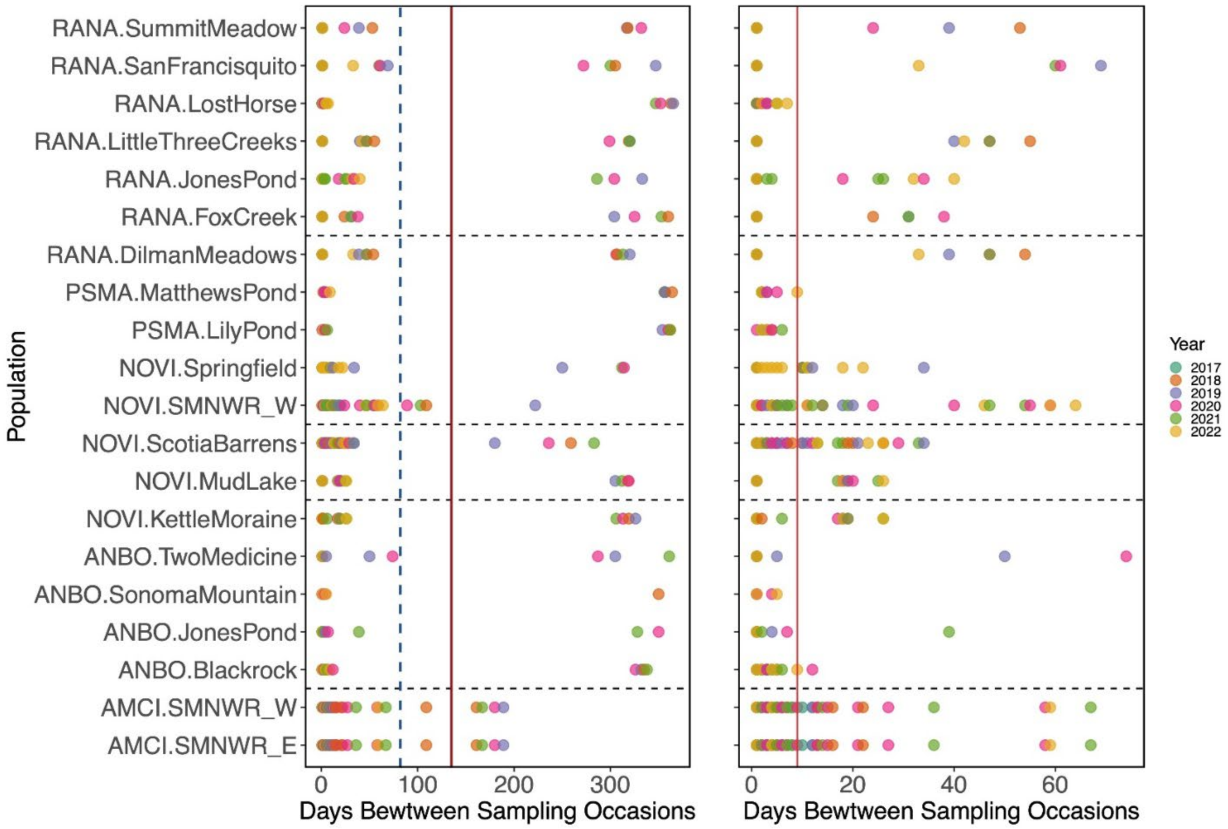

Figure S12. Gaps between consecutive sampling events in all populations. The left panel shows all events in all populations, the right panel zooms into all points to the left of (i.e., time gaps between sampling occasions less than) the blue dotted line in the left panel. Each point in each panel represents a single sampling occasion that occurred in a given year (color); the point's position along the X-axis indicates how many days elapsed between the previous sampling occasion and the plotted sampling occasion.

Left panel: The solid vertical red line and dotted blue lines give two proposed cutoffs in numbers of days by which to identify within- vs between-season gaps. That is, sampling events separated by more days than indicated by the blue dotted vertical line (or alternatively the dark red solid line) indicate sampling events that span an offseason (between which individual between-season survival [φ] will be estimated). Note that the only populations with sampling gaps between these lines are Florida populations. The blue dotted line was chosen for our analyses.

Right panel: The solid vertical red line shows the chosen threshold number of days between sampling events under which the population was assumed to be closed.

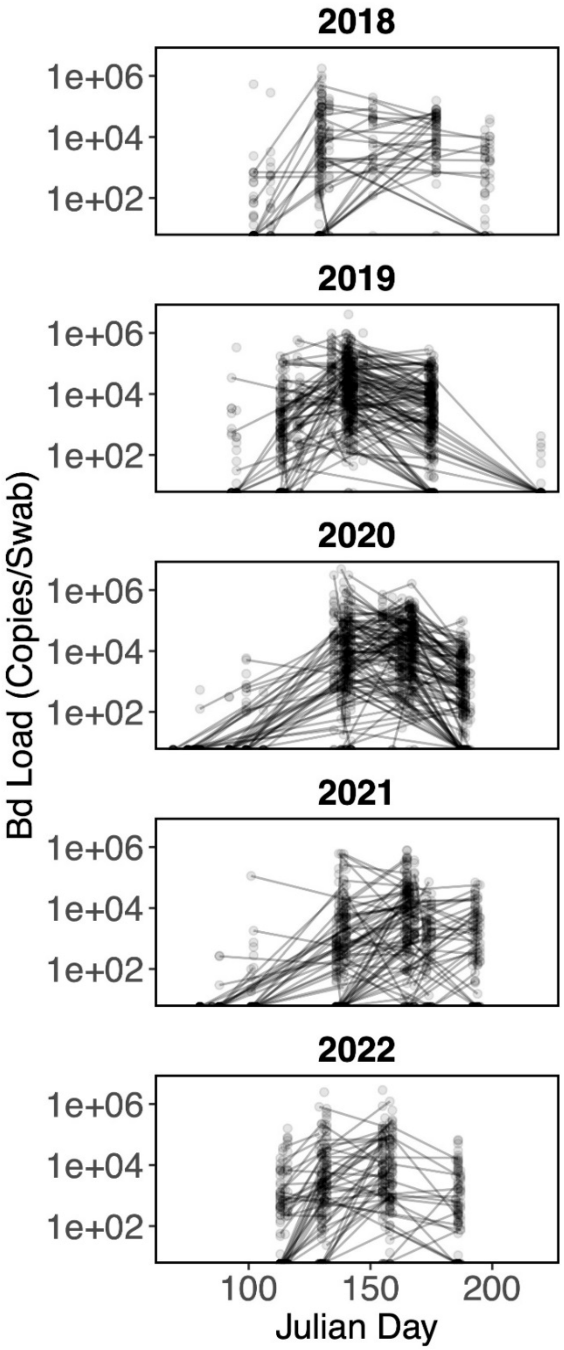

Figure S13. Detectable curvature in *Notophthalmus viridescens* Bd loads at the Scotia Barrens site, PA. Each point shows the load of an individual on a specific day; lines connect repeat measures within individuals within a year. All data pictured here are raw data, not modeled Bd loads. The y-axis is on a log scale.

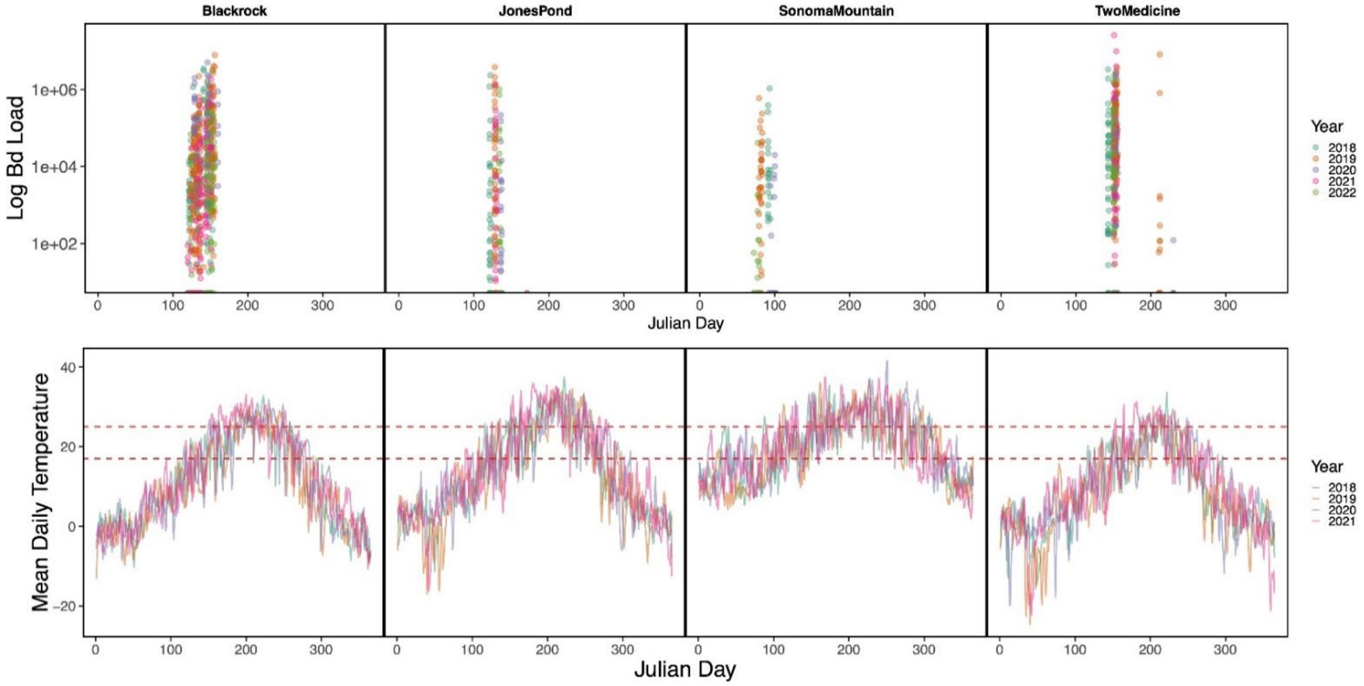

Figure S14. Bd loads (top row) and daily average temperatures throughout the year (bottom row) for all western toad populations. Each data point in the top panel represents a single swab in a single individual; Bd loads are presented on a log scale. Red dotted lines in the bottom panel envelop the *lab*-derived thermal optima (maximum growth rate) of Bd.

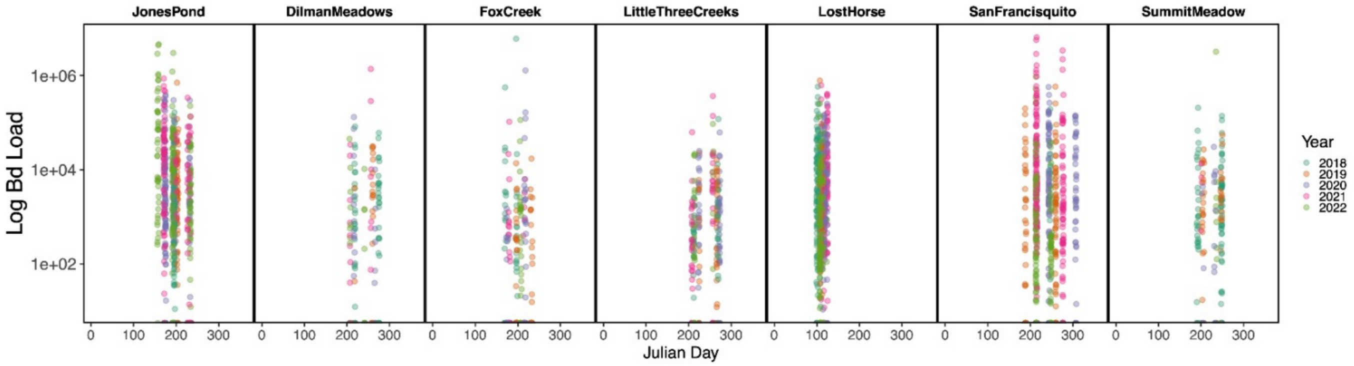

Figure S15. Bd load data from all populations of the *Rana* spp. show large variation among individuals on any given day, but little seasonal pattern in loads at a population level. Bd loads (y-axis) are presented on a log scale.

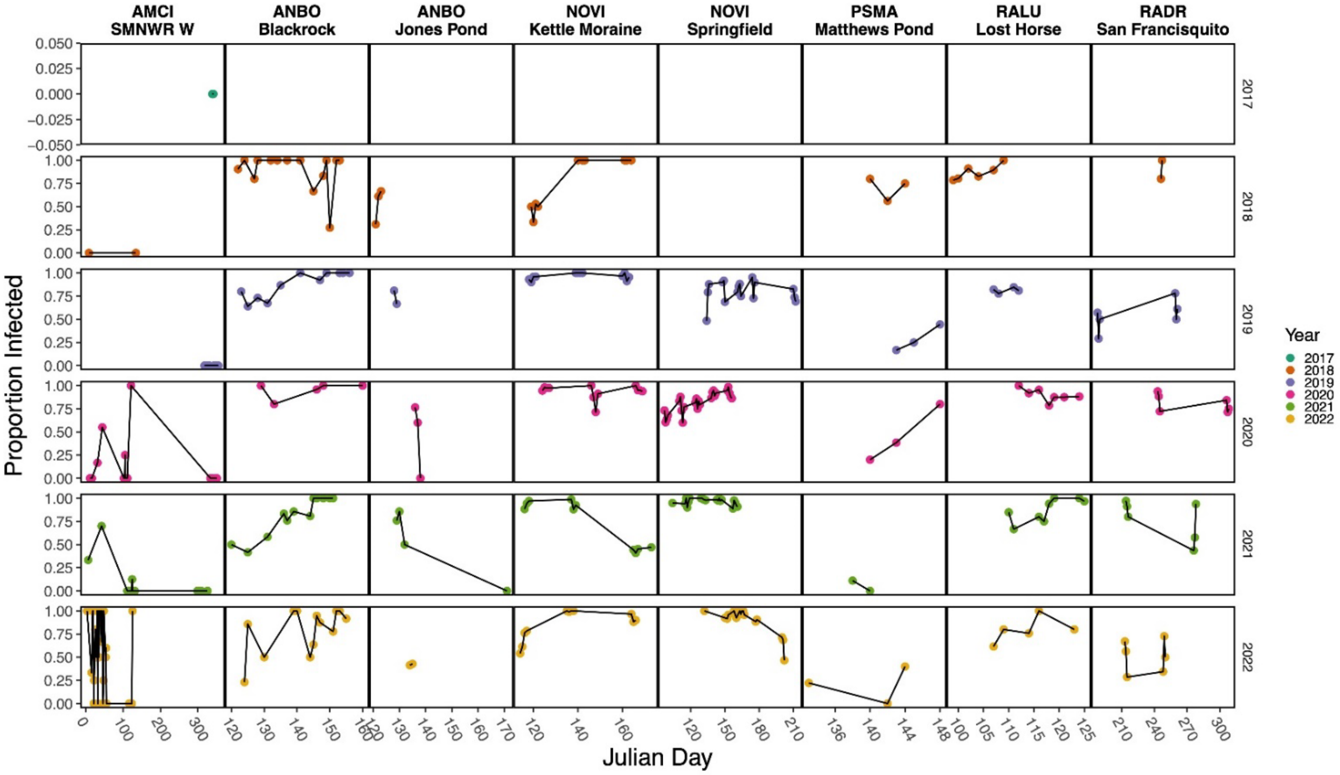

Figure S16. The proportion of captured individuals in an example 8 populations with detectably non-zero Bd loads. Each data point represents the total proportion of swabbed individual on a given day that tested positive for Bd.

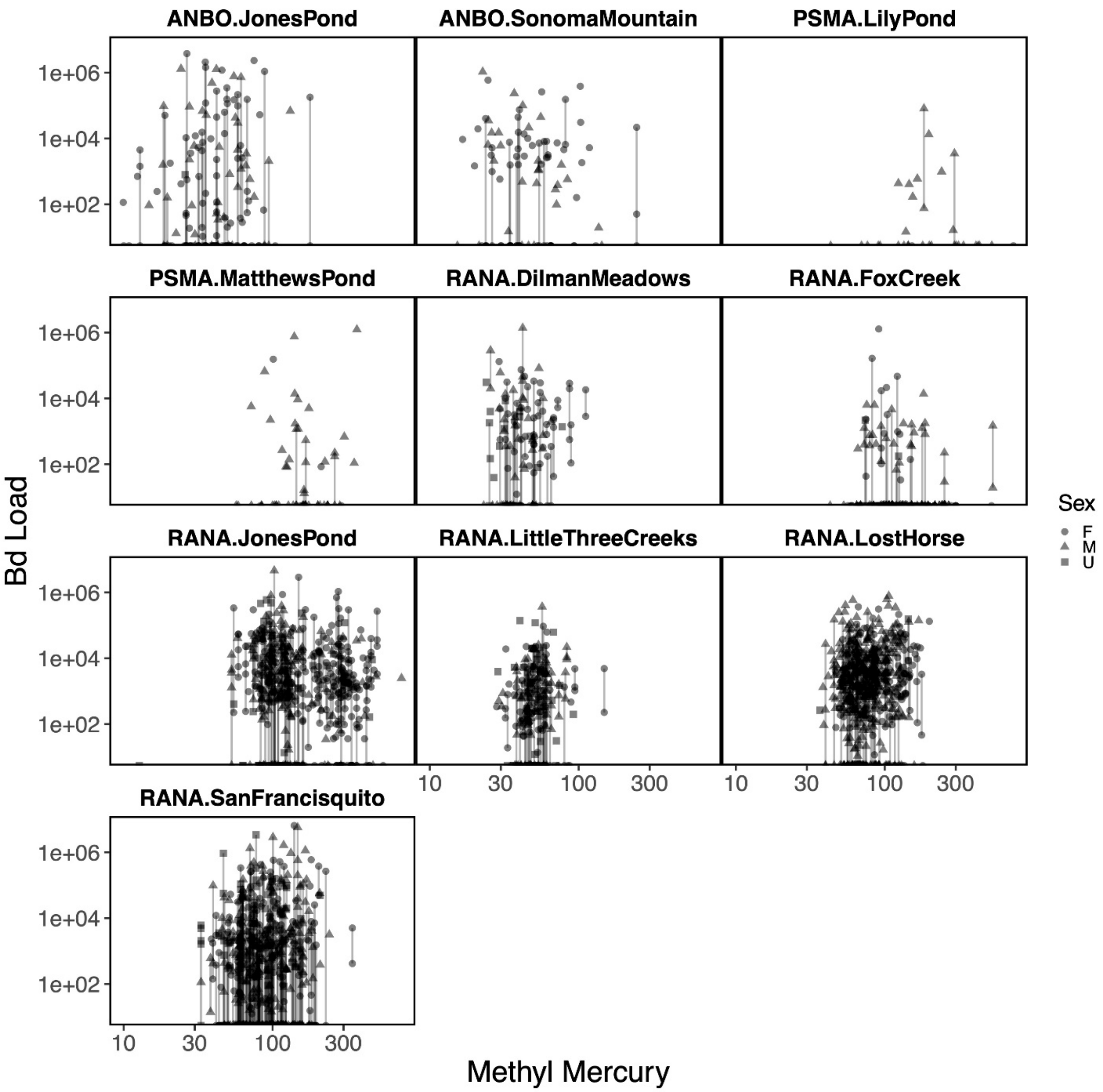

Figure S17. Methyl mercury (MeHg, x-axis) contamination is only very weakly correlated to Bd load. The 10 populations represented here are those fit using our primary model presented in the main text. Only animals with at least one Bd measure and an MeHg measure are plotted here (points); repeat Bd samples within the same individual are connected with vertical lines (individuals were only ever measured for MeHg one time). All data points are raw data; no imputed data are presented here. Both axes are on a log scale.

Supplemental Tables

Table S1: Data Quantity. Summary of available data in all sites.

Species	State	Site	Total # of Individuals Captured	Total # of Captures	% of Individuals Recaptured	Total # of Bd Swabs	% of Captured Individuals Swabbed	% of Individuals Recaptured After a Bd Swab	% of Individuals Measured For MeHg
AMCI	FL	SMNWR$_E$	373	415	0.09	163	0.42	0.03	0
AMCI	FL	SMNWR$_W$	289	333	0.11	228	0.69	0.07	0
ANBO	CA	Sonoma Mountain	101	330	0.65	127	0.99	0.65	0.73
ANBO	MT	Jones Pond	150	555	0.87	208	0.95	0.87	0.72
ANBO	MT	Two Medicine	602	909	0.31	391	0.58	0.2	0
ANBO	WY	Blackrock	825	1802	0.52	677	0.59	0.31	0.2
PSMA	CO	Lily Pond	365	523	0.33	119	0.3	0.12	0.14
PSMA	CO	Matthews Pond	269	364	0.24	127	0.42	0.1	0.27
RABO	CA	Fox Creek	306	601	0.49	379	0.92	0.46	0.42
RADR	CA	San Francisquito	578	1313	0.53	796	0.73	0.44	0.53
RASI	CA	Summit Meadow	232	546	0.58	180	0.64	0.41	0.05
RALU	MT	Jones Pond	464	1579	0.75	703	0.92	0.7	0.67
RALU	MT	Lost Horse	769	1421	0.52	804	0.89	0.46	0.64
RAPR	OR	Dilman Meadows	108	259	0.54	163	0.91	0.52	0.8
RACA	OR	Little Three Creeks	213	406	0.45	256	0.82	0.4	0.75
NOVI	FL	SMNWR$_W$	654	1106	0.32	684	0.69	0.24	0
NOVI	MA	Springfield	3009	4365	0.3	3755	0.89	0.27	0.01
NOVI	PA	Scotia Barrens	4368	9018	0.45	3472	0.54	0.27	0.1
NOVI	WI	Kettle Moraine	2405	2604	0.08	2503	0.96	0.07	0.09
NOVI	WI	Mud Lake	793	1012	0.22	999	0.99	0.22	0.16

Table S2: Sampling Occasions. Number of sampling occasions (left number in each cell). Bracketed numbers in each cell give the number of calendar months in which sampling occurred in each year (note that a 2 does not necessarily mean that sampling spanned ~ 60 days, just that sampling occurred in two different calendar months).

**Species**	**State**	**Site**	**2017**	**2018**	**2019**	**2020**	**2021**	**2022**
Ambystoma cingulatum	FL	SMNWR$_E$	28 [3]	57 [8]	23 [5]	23 [7]	41 [7]	66 [4]
Ambystoma cingulatum	FL	SMNWR$_W$	28 [3]	57 [8]	23 [5]	23 [7]	41 [7]	66 [4]
Anaxyrus boreas	CA	Sonoma Mountain		4 [1]	5 [1]	7 [1]	0 [0]	7 [1]
Anaxyrus boreas	MT	Jones Pond		3 [1]	3 [1]	4 [1]	4 [2]	3 [1]
Anaxyrus boreas	MT	Two Medicine		3 [1]	7 [3]	6 [2]	4 [1]	4 [2]
Anaxyrus boreas	WY	Blackrock		15 [2]	15 [2]	11 [2]	14 [2]	17 [2]
Notophthalmus viridescens	FL	SMNWR$_W$		3 [1]	15 [3]	61 [9]	63 [9]	77 [7]
Notophthalmus viridescens	MA	Springfield			17 [4]	24 [3]	21 [3]	29 [4]
Notophthalmus viridescens	PA	Scotia Barrens		13 [4]	42 [6]	52 [5]	29 [5]	15 [4]
Notophthalmus viridescens	WI	Kettle Moraine		11 [3]	12 [3]	12 [2]	10 [3]	11 [3]
Notophthalmus viridescens	WI	Mud Lake		12 [2]	11 [2]	11 [3]	12 [3]	11 [3]
Pseudacris maculata	CO	Lily Pond		3 [2]	3 [1]	3 [1]	3 [2]	3 [2]
Pseudacris maculate	CO	Matthews Pond		3 [1]	3 [1]	3 [1]	3 [1]	3 [1]
Rana boylii	CA	Fox Creek		10 [2]	10 [2]	10 [2]	10 [2]	5 [1]
Rana cascadae	OR	Little Three Creeks		6 [3]	6 [2]	6 [3]	6 [2]	5 [2]
Rana draytonii	CA	San Francisquito		3 [1]	6 [2]	6 [3]	6 [2]	6 [2]
Rana luteiventris	MT	Jones Pond		3 [1]	4 [1]	11 [3]	11 [3]	10 [3]
Rana luteiventris	MT	Lost Horse		6 [1]	4 [1]	7 [2]	8 [2]	5 [2]
Rana pretiosa	OR	Dilman Meadows		6 [2]	6 [2]	6 [2]	6 [2]	5 [2]
Rana sierrae	CA	Summit Meadow		9 [2]	10 [2]	10 [2]	4 [1]	3 [1]

Table S3: Captures. Number of individuals captured in each year (left number in each cell). The bracketed number in each cell gives the total number of individuals that were recaptured at least one time in that calendar year. Note that these numbers do not indicate recaptures across years.

Species	State	Site	2017	2018	2019	2020	2021	2022
Ambystoma cingulatum	FL	SMNWR$_E$	84 [17]	174 [7]	15 [1]	17 [1]	46 [0]	49 [1]
Ambystoma cingulatum	FL	SMNWR$_W$	27 [3]	54 [1]	56 [2]	50 [2]	47 [0]	74 [9]
Anaxyrus boreas	CA	Sonoma Mountain		22 [5]	42 [19]	24 [20]		37 [33]
Anaxyrus boreas	MT	Jones Pond		64 [59]	50 [41]	28 [23]	39 [33]	36 [32]
Anaxyrus boreas	MT	Two Medicine		182 [2]	158 [23]	178 [48]	78 [18]	150 [45]
Anaxyrus boreas	WY	Blackrock		282 [121]	198 [104]	199 [67]	234 [105]	201 [65]
Notophthalmus viridescens	FL	SMNWR$_W$		8 [0]	159 [28]	278 [95]	173 [52]	136 [37]
Notophthalmus viridescens	MA	Springfield			495 [75]	1230 [259]	999 [143]	807 [163]
Notophthalmus viridescens	PA	Scotia Barrens		505 [160]	1726 [671]	1796 [675]	1134 [312]	462 [93]
Notophthalmus viridescens	WI	Kettle Moraine		213 [14]	512 [12]	463 [39]	673 [28]	604 [38]
Notophthalmus viridescens	WI	Mud Lake		167 [20]	232 [26]	184 [19]	194 [41]	95 [22]
Pseudacris maculata	CO	Lily Pond		64 [3]	101 [10]	94 [10]	89 [16]	108 [20]
Pseudacris maculata	CO	Matthews Pond		73 [6]	36 [7]	71 [4]	77 [10]	75 [3]
Rana boylii	CA	Fox Creek		83 [27]	119 [41]	85 [25]	67 [32]	49 [15]
Rana cascadae	OR	Little Three Creeks		70 [11]	84 [27]	56 [17]	49 [7]	50 [13]
Rana draytonii	CA	San Francisquito		225 [45]	124 [42]	110 [30]	217 [102]	175 [88]
Rana luteiventris	MT	Jones Pond		87 [37]	61 [50]	147 [117]	117 [86]	147 [113]
Rana luteiventris	MT	Lost Horse		172 [81]	122 [46]	171 [68]	174 [72]	243 [122]
Rana pretiosa	OR	Dilman Meadows		63 [23]	27 [11]	23 [16]	13 [10]	18 [13]
Rana sierrae	CA	Summit Meadow		120 [62]	54 [34]	49 [21]	16 [10]	58 [22]

Table S4: State, federal (ESA-listed species only), and other permits and animal care and use protocols for each site and species included in the study.

Region	PI	MeHg	CMR (2018-2022)	Bsal (2017 and prior)	USFWS	State
RMN	Hossack	003-18BHW-020618	003-18BHW-020618	IACUC-Muths_27Mar2014	NA	NA
NOCAL	Halstead	WERC-2014-01	WERC-2014-01	WERC-2014-01	TE-844852	SCP10779
PNW	Adams	ACUP 2021-001	ACUP 2021-001	ACUP 2015-003a	TE49790B-3	075-20, 049-21, 049-22
SOCAL	Fisher	USGS_IACUC_WERC_FS_2023_02	USGS_IACUC_WERC_FS_2023_02	USGS_IACUC_WERC_FS_2023_02	TE - 045994-19	SCP838
MW	Grear	EP170227	EP170227	EP160329 (KY), EP170227 (NWHC)	NA	NA
NE	Grant	2012-02P(Patuxent)	2012-02P(Patuxent)	2012-02P(Patuxent)	NA	NA
NE	Miller	IACUC 45187*	IACUC 45187	2012-02P(Patuxent)	NA	NA
RMS	Muths	FORT IACUC2013-09	FORT IACUC2013-09	IACUC-Muths_27Mar2013	NA	NA
SC	Waddle	USGS/WARC/LFT 2019-03	USGS/WARC/LFT 2019-03	USGS/WARC/LFT 08-01	NA	NA
